# High genome heterozygosity revealed vegetative propagation over the sea in Moso bamboo

**DOI:** 10.1186/s12864-023-09428-9

**Published:** 2023-06-24

**Authors:** Norihide Nishiyama, Akihisa Shinozawa, Takashi Matsumoto, Takeshi Izawa

**Affiliations:** 1 Department of Agricultural and Environmental Biology, Laboratory of Plant Breeding & Genetics, The University of Tokyo, 1-1-1 Yayoi, Bunkyo-Ku, Tokyo, 113-8657 Japan; 2 Department of Bioscience, Faculty of Life Sciences, Tokyo University of Agriculture, 1-1-1 Sakuragaoka Setagaya-Ku, Tokyo, 156-8502 Japan; 3 The NODAI Genome Research Center (NGRC), Tokyo University of Agriculture, 1-1-1 Sakuragaoka Setagaya-Ku, Tokyo, 156-8502 Japan

**Keywords:** Moso bamboo, Kikko-chiku, GRAS-Di, Heterozygosity, Vegetative propagation

## Abstract

**Background:**

Moso bamboo (*Phyllostachys edulis*) is a typical East Asian bamboo that does not flower for > 60 years and propagates without seed reproduction. Thus, Moso bamboo can be propagated vegetatively, possibly resulting in highly heterozygous genetic inheritance. Recently, a draft genome of Moso bamboo was reported, followed by whole genome single nucleotide polymorphisms (SNP) analysis, which showed that the genome of Moso bamboo in China has regional characteristics. Moso bamboo in Japan is thought to have been introduced from China over the sea in 1736. However, it is unclear where and how Moso bamboo was introduced in Japan from China. Here, based on detailed analysis of heterozygosity in genome diversity, we estimate the spread of genome diversity and its pedigree of Moso bamboo.

**Results:**

We sequenced the whole genome of Moso bamboo in Japan and compared them with data reported previously from 15 regions of China. Only 4.1 million loci (0.37% of the analyzed genomic region) were identified as polymorphic loci. We next narrowed down the number of polymorphic loci using several filters and extracted more reliable SNPs. Among the 414,952 high-quality SNPs, 319,431 (77%) loci were identified as heterozygous common to all tested samples. The result suggested that all tested samples were clones via vegetative reproduction. Somatic mutations may accumulate in a heterozygous manner within a single clone. We examined common heterozygous loci between samples from Japan and elsewhere, from which we inferred that an individual closely related to the sample from Fujian, China, was introduced to Japan across the sea without seed reproduction. In addition, we collected 16 samples from four nearby bamboo forests in Japan and performed SNP and insertion/deletion analyses using a genotyping by sequencing (GBS) method. The results suggested that a small number of somatic mutations would spread within and between bamboo groves.

**Conclusions:**

High heterozygosity in the genome-wide diversity of Moso bamboo implies the vegetative propagation of Moso bamboo from China to Japan, the pedigree of Moso bamboo in Japan, and becomes a useful marker to approach the spread of genome diversity in clonal plants.

**Supplementary Information:**

The online version contains supplementary material available at 10.1186/s12864-023-09428-9.

## Background

Many bamboo species reproduce vegetatively for several decades and rarely flower [1]. Moso bamboo (*Phyllostachys edulis*) is a typical East Asian bamboo with about 4.43 million hectares of habitat in China, accounting for about 73.8% of China’s bamboo forest area [2]. Moso bamboo has been reported to flower once after an interval of > 60 years, but the flowering cycle and mechanism are unknown [3–7].

A small number of DNA markers have been used to analyze genomic diversity within Moso bamboo. In Taiwan, nine strains of Moso bamboo were distinguished using random amplified polymorphic DNA (RAPD), microsatellite, and minisatellite markers [8]. In China, analyses using 20 simple sequence repeat (SSR) markers showed that Moso bamboo from three regions was polymorphic within the region [9]. Analysis of 16 microsatellite markers revealed that all 246 strains of Moso bamboo in China and Japan had identical genotypes. Four of the markers were heterozygous, suggesting that a single clone is widely distributed in East Asia, including Japan [10]. However, it is difficult to realize the genomic diversity of Moso bamboo based on these markers alone.

A draft genome of Moso bamboo of about 2 Gb was recently reported [11]. Subsequently, a reference genome with chromosome-level scaffolds was created using Hi-C [12], and then used as the reference genome for whole-genome single nucleotide polymorphism (SNP) analysis of 427 strains of Moso bamboo from various regions of China. By clustering using the neighbor-joining (NJ) method [13], Hunan Province was inferred to be the origin of Moso bamboo because the bamboo in this area is close to *Phyllostachys kwangsiensis*, and its Wright’s F statistic (*F*st) is larger than that of other regions. In addition, Moso bamboo from Guizhou and Yunnan provinces were inferred to be recent isolates from eastern China, based on their low genetic diversity and clustering results [14].

Moso bamboo in Japan is thought to have been introduced to Kagoshima, the second most southern prefecture in Japan, from China over the sea in 1736 [15]. Although there may have been other introductions of Moso bamboo from China to Japan [16, 17], there is insufficient evidence to confirm this. A stone monument in Senganen Garden, Kagoshima Prefecture, explicitly states that Moso bamboo was brought to Japan in 1736. Since Moso bamboo shoot is used as food, and the culm is also a useful material, it is thought that the distribution of Moso bamboo greatly expanded in Japan due to human cultivation. Senganen is in the southern part of Japan (31°4 N), but the current northern limit of Moso bamboo in Japan is around Hakodate (41°5 N).

It is not possible to determine whether Moso bamboo was brought from China as seeds or plant bodies based on the description on the Senganen stone monument, which also gives no indication of the region in China from which Moso bamboo was brought to Japan. Here, in this study, we inferred the origin of Japanese Moso bamboo by whole-genome resequencing (WGS) analysis of specimens from Japan and China. We used a lineage of Japanese Moso bamboo that had been transplanted from Senganen to Fuji Bamboo Garden in Shizuoka Prefecture. We also sampled kikko-chiku (*Phyllostachys edulis* f. *heterocycla*) (Additional file 1: Fig. S1), which is thought to be a variety of Moso bamboo, in Shizuoka Prefecture, Japan, and investigated its origin.

To further elucidate the mechanisms underlying the diversity of Moso bamboo, a recently developed genotyping by sequencing (GBS) method, GRAS-Di (Genotyping by Random Amplicon Sequencing-Direct) [18], was used to analyze multiple samples from the same bamboo grove in Japan and other, nearby groves. An overview of the WGS and GRAS-Di analysis methods, and a description of their use in this study, is presented in Additional file 1: Fig. S2.

## Results

### In the WGS analysis, the majority of high-quality SNPs were identified as heterozygous loci among all tested samples

**Fig. 1:**
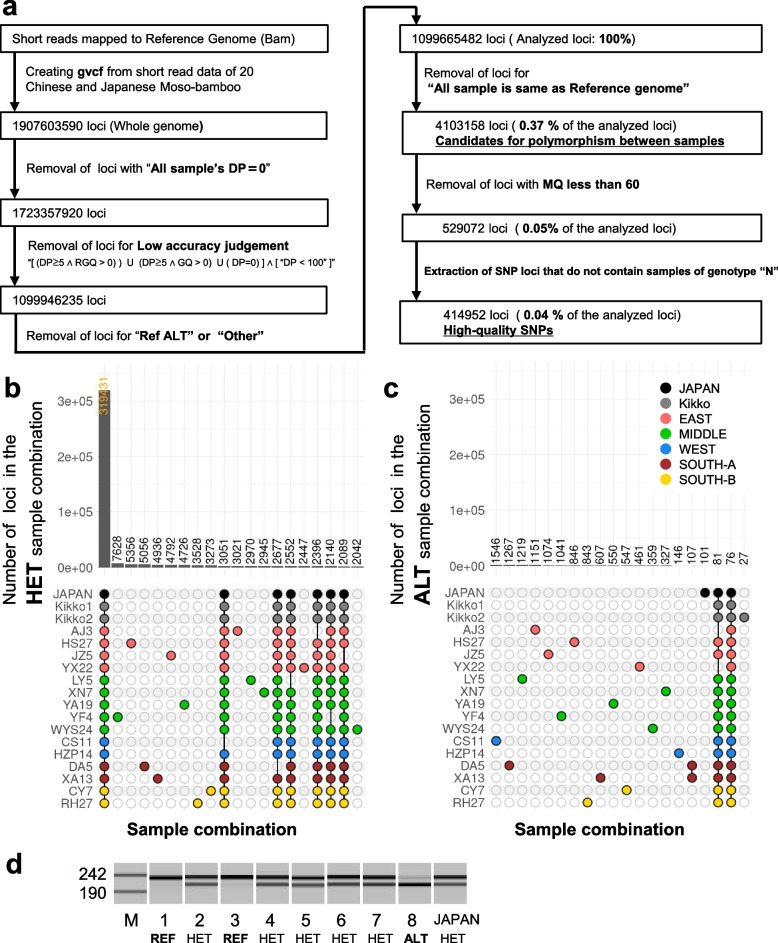
The majority of the high-quality SNPs were heterozygous loci among all tested samples. **a** High-quality SNPs were selected in several steps. “gvcf” contains records for all sites (with or without mutation detection). DP, read depth; GQ, genotype quality; RGQ, GQ at reference sites; MQ, root mean square of the mapping quality of reads across all samples; N, no reads at the site; Ref ALT, loci determined to be homozygous alternative when a variant call was made for the short read used to create the reference genome; Other, loci that were not in the homozygous reference, heterozygous, or homozygous alternative category. GATK-4.2.3 HaplotypeCaller was used for variant calling, with the -ERC BP_RESOLUTION option. **b, c** High-quality SNPs were analyzed using UpSet plots. Many loci were heterozygous in all samples tested. These plots were created using the R package ComplexUpset. The top 20 combinations in terms of the number of loci were arranged in descending order. Combinations without at least one sample were excluded. The classification of five regions of China (East, Middle, West, South-A, and South-B) is the same as a previous report [14]. **b** Heterozygous sample combinations and the number of loci. HET, heterozygous. **c** Homozygous alternative sample combinations and the number of loci. ALT, homozygous alternative. **d** Loci where all tested samples were heterozygous in WGS analysis were not heterozygous in some of the seedlings of Moso bamboo obtained in 2022. Samples from 1 to 8 were seedlings from different seeds. JAPAN, Japanese Moso bamboo analyzed by WGS; M, pUC19 DNA/MspI (HpaII) Marker. We used dCAPs to detect polymorphisms. 228 bases around scaffold7:10,257,001 were amplified using PCR. After that, restriction enzyme treatment was performed with SmaI. The terminal 23 bases were cleaved by SmaI only in the case of the sequence where scaffold7:10,257,001 was an alternative. Electrophoresis was performed using DNA-500 with Multina. REF, homozygous reference; HET, heterozygous; ALT, homozygous alternative

We used Genome Analysis Toolkit (GATK) 4.2.3.0 [19] for variant-calling of 20 whole-genome short-read data, including 1 lineage of Moso bamboo thought to have been first introduced to Japan from China, 2 lineages of Japanese kikko-chiku thought to be a variant of Moso bamboo, and 15 lineages from various regions in China [14], and 125- and 250-bp paired-end short read data [12] used to construct the reference genome of Moso bamboo. For the 15 Chinese lineages, one canonical representative from each cluster was selected based on principal component analysis (PCA) conducted in a previous report [14]. Referring to PCA data from the previous report [14], we calculated the median PC1, PC2, and PC3 for each of the 15 regions and selected one sample in each region with the lowest Euclidean distance between the sample and the median PC1, PC2, and PC3 for that sample's region. In this paper, the classification of five regions of China (East, Middle, West, South-A, and South-B) is the same as a previous report [14]. For whole-genome analysis using highly reliable loci, a gvcf file containing genotyping data for all nucleotide loci in the whole reference genome was created, followed by filtering to remove loci with low genotyping reliability. Loci identified as homozygous alternatives in the variant calling using the Illumina short-read data used to create the reference genome were excluded due to possible sequence errors in the reference genome. In total, 4,103,158 loci (0.37% of the analyzed sequences) were candidate loci with polymorphisms. 99.63% of the sequences in the analyzed region were homozygous reference sequences. As Moso bamboo propagates in a vegetative manner [6, 10], information on heterozygous loci was considered. High-quality SNPs were selected to infer phylogenetic relationships. We used the MQ value (root mean square of the mapping quality of reads across all samples) to narrow down the candidate SNPs to only the most reliable ones; the highest MQ value (corresponding to the highest genotyping accuracy) was 60 (Fig. 1a). The loci with MQ values less than 60 were widely distributed and these were excluded (Additional file 1: Fig. S3). The 414,952 loci extracted by filtering by MQ value were used as high-quality SNPs in the following analysis. We investigated common heterozygous and homozygous loci among 18 samples, except for the 125- and 250 bp paired-end short read data when the reference genome was created. Using the R package complex-upset v1.3.3 [20], the relationship between samples was illustrated by a method called UpSet plots [21]. Among the 414,952 high-quality SNPs remaining after the selection process, 319,431 (77%) loci were identified as heterozygous among all 18 samples tested in common, and there were only 76 homozygous alternative loci among all 18 samples tested in common (Fig. 1b, c). During nutritional reproduction; homozygous alternative loci were considered unlikely to occur. A report examining mutations during trophic reproduction in several plants found that 99% of mutations were heterozygous [22]. However, a small number of loci containing some homozygous alternative samples were in fact detected (Fig. 1c). Of the high-quality SNPs, 3.1% (12,923/414,952 loci) contained samples of the homozygous alternative, of which 99.4% (12,847/12,923 loci) contained homozygous reference and/or heterozygous samples were included (Additional file 1: Fig. S4a). 89.2% of the loci (11,459/12,847 loci) had one homozygous alternative sample; the other samples were heterozygous (Additional file 1: Fig. S4b, c).Therefore, we speculated that a small number of the homozygous alternative loci may have arisen from heterozygous rather than homozygous reference loci. It is possible for a heterozygous locus caused by a somatic mutation to become a homozygous alternative locus via a double-strand break and homologous recombination (HR) (Additional file 1: Fig. S5).

### High-quality SNPs that were heterozygous in all tested samples in the WGS analysis were biased in the genome

We examined the genomic distribution of 414,952 high-quality SNPs and their correspondence to genomic mappabilty using GenMap [23]. As a result, the distribution of high-quality SNPs in the 414,952 loci was somewhat skewed, not linked to the genomic mappabilty, but the 97,869 loci, excluding loci for which all samples were heterozygous or all samples were homozygous alternatives, were evenly distributed throughout the genome (Additional file 1: Fig. S6a, b). The number of loci for which all samples were homozygous alternative was only 76 (Fig. 1c). Therefore, the majority of the bias of the distribution of 414,952 high-quality SNPs on the genome was caused by heterozygous loci in all tested samples. To investigate the certainty of this result, it was examined whether genotyping of loci, in which all 18 samples were heterozygous, was performed correctly. Heterozygous loci genotyping might be considered homozygous genotyping if the depth of reads is insufficient, for example. Therefore, of the 319,431 loci for which all samples were heterozygous, 150 loci from the scaffold1 end were visually inspected using the Integrative Genomics Viewer (IGV) v2.11.1 [24]. The results of visual inspection using IGV and genotyping by GATK were in agreement (Additional file 2: Table S1), suggesting that genotyping by GATK after filtering was reliable in most cases. We also examined the 8 loci that were heterozygous in all samples in the WGS analysis using Sanger sequencing of Japanese Moso bamboo, for which we performed WGS analysis. For chromosome-level scaffolds 1–8, one arbitrary SNP was selected for each so that linkage need not be considered. The results were identical to the WGS results and all were heterozygous (Additional file 3: Table S2). In the case of seedling-derived Moso bamboo [7] from 8 different seeds, 4 of the 8 loci were examined by dCAPs [25] and restriction enzyme treatments, and in some case of seedling-derived Moso bamboo, they turned out to be homozygous references or homozygous alternatives. (Fig. 1d; Additional file 3: Table S2). This result made sense; According to Mendel's Law of Segregation [26], when sexual reproduction occurs, many loci that were heterozygous in the parent become homozygous in the offspring, whether self or outcross (Additional file 1: Fig. S7). We, then, performed WGS analysis on the Moso bamboo thought to have been first introduced to Japan and the 8 seedlings, following the same flow as we did for the WGS analysis involving samples from 15 regions of China (Additional file 1: Fig. S8a, b). As a result, many of the heterozygous loci in Moso bamboo thought to have been first introduced to Japan were replaced by homozygous reference or homozygous alternative in the 8 seedlings (Additional file 1: Fig. S8c, d). As for the heterozygous loci, if 2 times self-pollinations occur and progenies are created, the homozygous reference: heterozygous: homozygous alternative is likely to have a separation ratio of 3:2:3 in the progenies (Additional file 1: Fig. S8a). The actual segregation ratio in the 8 individual seedlings was relatively close to such a segregation ratio (Additional file 1: Fig. S8d). We found that the heterozygous loci in Japanese Moso bamboo first introduced to Japan were fixed to the homozygous reference or homozygous alternative in a relatively large area in the 8 seedlings. We also found both homozygous and heterozygous regions in a single scaffold in the 8 seedlings, suggesting that homologous recombination had occurred (Additional file 1: Fig. S8e). In the 8 seedlings, a homozygous reference and a homozygous alternative were found in close proximity (Additonal file 1: Fig. S8e). We speculated that the reference genome of Moso bamboo [12] is a complex combination of 2 haplotypes because the Moso bamboo reference genome was generated using short read sequencing (Additional file 1: Fig. S8f). The majority of heterozygous loci in Japanese Moso bamboo thought to be first introduced to Japan also coincided with heterozygous loci in Moso bamboo from 15 regions of China (Additional file 1: Fig. S9a,b,c). These results suggest that the Moso bamboo in Japan except seedlings, China and kikko-chiku in Japan tested in this study are a single clone, and that the Moso bamboo in Japan was introduced from China as trophically reproduced individuals rather than seeds. In addition, since the presence of heterozygous regions unevenly distributed in the genome (Additional file 1: Figs. S6a,b, S9c), we speculated that Moso bamboo which is widely distributed from China to Japan, is a lineage derived from sexual reproduction between the same species in the genus *Phyllostachys* or between different closely related species in the genus *Phyllostachys*.

### Clustering analysis and PCA using high-quality SNPs derived from WGS analysis showed that the genome could be characterized by region, but the origin of Japanese Moso bamboo was not clear

**Fig. 2:**
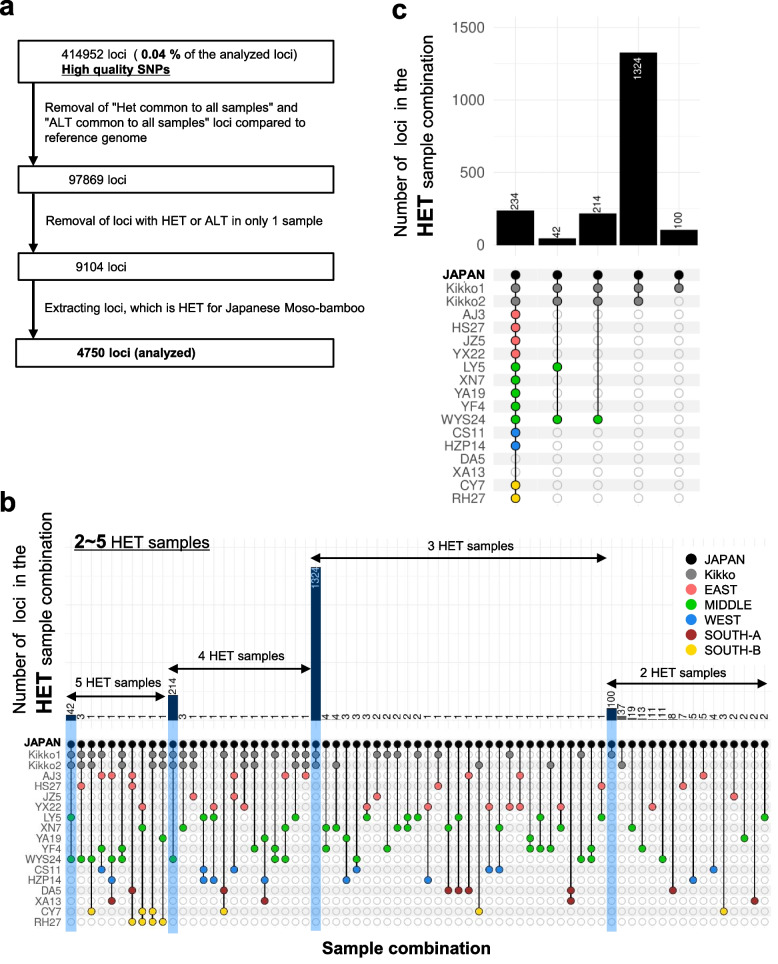
Heterozygous loci common among samples were analyzed. UpSet plots were used to determine the origin of Japanese Moso bamboo. **a** To examine the phylogenetic relationships among samples, loci with no polymorphisms among samples or one sample having a different genotype from the others were excluded. Of the remaining loci, only those for which Japanese Moso bamboo is heterozygous were selected. **b, c** The 4,750 selected SNPs were analyzed using UpSet plots. The classification of five regions of China (East, Middle, West, South-A, and South-B) is the same as a previous report [14]. **b** All combinations of loci with ≤ 5 heterozygous samples are shown. Sample combinations are presented in descending order of loci. Analysis was performed with the sort_intersections_by = c (‘degree’,‘cardinality’) option selected. For loci combinations with ≥ 6 heterozygous samples, see Additional file 1: Fig. S11. The number of loci in the light blue background sample combination was significantly higher than in other sample combinations with the same number of heterozygous samples. **c** Based on the results of the analysis shown in Fig. 2b and Additional file 1: Fig. S11, only sample combinations with a significantly high number of loci (considered to reflect phylogenetic relationships between samples) were extracted. HET, heterozygous; ALT, homozygous alternative

To infer phylogenetic relationships among samples analyzed by WGS, it is necessary to analyze polymorphic loci among samples. Therefore, among the high-quality SNPs, we first excluded loci that differed from the reference genome but were not polymorphic among samples, i.e., heterozygous or homozygous alternative loci among all tested samples; 414,952 high-quality SNPs were narrowed down to 97,869 in this manner. The presence of a SNP in only one sample among all those tested indicates that the sample is different from the others, but is not suitable for estimating phylogenetic relationships. Therefore, we also removed loci including only one heterozygous or homozygous alternative sample, after which 9,104 loci remained (Fig. 2a). Clustering was performed using pvclust v2.2.0 [27]. Clustering analysis of the 9,104 loci yielded clusters for each region of China, and for Japanese Moso bamboo and kikko-chiku, but the origin of the Japanese Moso bamboo remained unclear (Additional file 1: Fig. S10a). Similarly, PCA showed the genomic characteristics by region, but the origin of Japanese Moso bamboo was still unclear. In principal components (PCs) 1 and 2, samples from the Middle region of China were close to Japanese Moso bamboo, while in PC3 and PC4, samples from the West region were close to Japanese Moso bamboo (Additional file 1: Fig. S10b).

### WGS analysis of common heterozygous loci among samples in high-quality SNPs allowed us to infer the origin of Japanese Moso bamboo

**Fig. 3:**
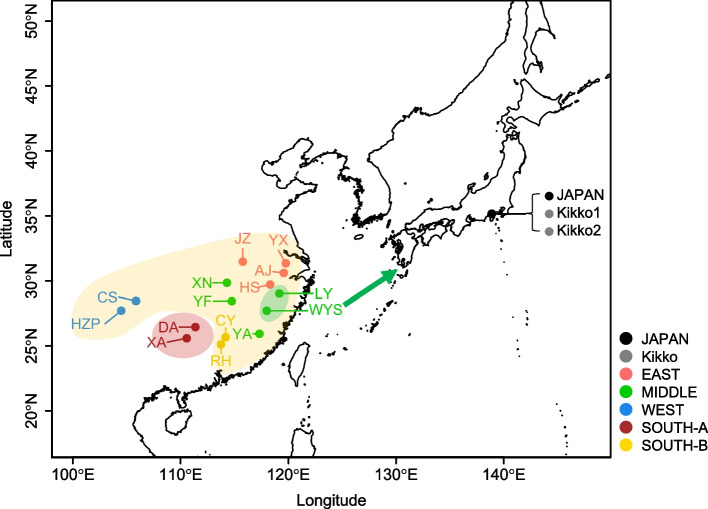
Map showing the phylogenetic relationships between Chinese and Japanese Moso bamboo. It was assumed that South-A region (DA, XA) in China diverged from other samples, after which the samples in part of the Middle region diverged from the other samples. The classification of five regions of China (East, Middle, West, South-A, and South-B) is the same as a previous report [14]. We estimate that the Moso bamboo around the WYS region (in the Middle region) was introduced into Japan over the sea in 1736. It is further speculated that a somatic mutation subsequently occurred in Japanese Moso bamboo, after which Japanese kikko-chiku appeared

We then devised an alternative method to clustering analysis and PCA to estimate the origin of Japanese Moso bamboo. We assumed that all tested samples were trophic individuals. In this case, polymorphisms among samples would likely be caused by the accumulation of novel heterozygous somatic mutations. Therefore, we hypothesized that phylogenetic relationships could be found by examining common heterozygous loci among tested samples. To estimate the origin of Japanese Moso bamboo, we extracted 4,750 loci from among the 9,104 where the Moso bamboo in Japan is heterozygous (Fig. 2a), and identified those that were common among the samples. We used UpSet plots [21], which are useful for illustrating complex relationships, to determine the number of SNPs in combinations of common heterozygous loci (Fig. 2b, c; Additional file 1: Fig. S11). In UpSet plots, sample combinations with an “outlier peak” may indicate a phylogenetic relationship. Sample combinations with small numbers of SNPs may contain genotyping errors. We estimated that Moso bamboo first diverged into a population that included Japanese and Chinese South-A (XA13:Guangxi and DA5: Hunan) subpopulations, after which the Japanese Moso bamboo-containing population diverged into the LY (Zhejiang) and WYS (Fujian) regions, and other populations. A lineage of Moso bamboo closely related to the WYS sample was then introduced into Japan. As many heterozygous loci common only to Japanese kikko-chiku and Japanese Moso bamboo were found, we estimated that the kikko-chiku in Japan most likely originated from Moso bamboo in Japan (Figs. 2b, c and 3, Additional file 1: Fig. S11). We further investigated why the phylogenetic relationship between the Japanese and Chinese samples was not clear in the clustering and PCA (Additional file 1: fig. S10a, b). Analysis using the UpSet plot inferred a very close phylogenetic relationship between Japanese Moso bamboo and the 2 samples of Japanese kikko-chiku (Fig. 2b, c). Therefore, we hypothesized that in the manipulation excluding only one sample with a heterozygous loci or only one sample with a homozygous alternative loci (Fig. 2a), SNPs characteristic of Japanese Moso bamboo and kikko-chiku would be selected so much. It could consequently affect the results of clustering and PCA. Therefore, we performed a clustering analysis similar to Fig. S10a, except for the 2 kikko-chiku samples from Japan, and found that, similar to the results of the UpSet plot analysis (Fig. 2b, c; Additional file 1: Fig. S11), among the Chinese samples, WYS24 was estimated to be the closest lineage to the Japanese Moso bamboo (Additional file 1: Fig. S12). When estimating phylogenetic relationships by clustering a small number of samples, the bias of the selected samples may have a greater impact on the results of the analysis. Even with the clustering analysis excluding the 2 Japanese kikko-chiku samples, unlike the UpSet plot analysis, we could not estimate that LY5 is the next most closely related sample to the Japanese Moso bamboo after WYS24 (Additional file 1: Fig. S12). Our proposed method of estimating phylogenetic relationships using UpSet plot analysis is an effective method for phylogenetic analysis was less subject to bias in the selection of samples and more effective in clarifying phylogenetic relationships.

### Genomic diversity of Moso bamboo in Japan and the mechanisms that generate the diversity

**Fig. 4:**
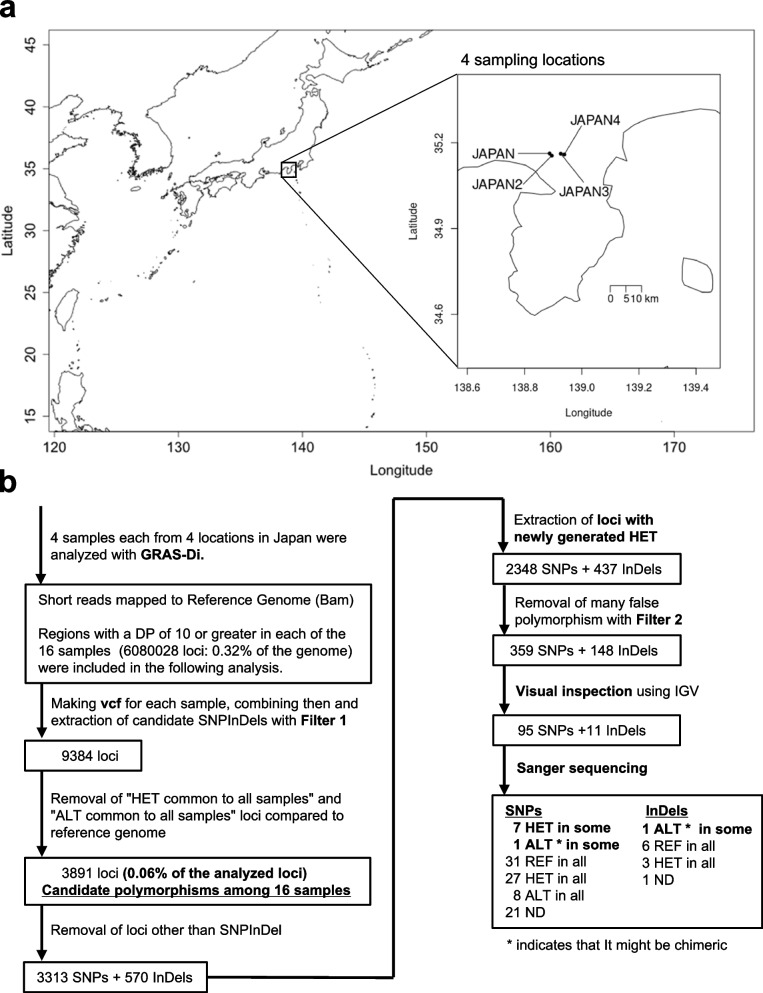
Moso bamboo diversity was studied within the same bamboo forest, and among neighboring bamboo forests. **a** Moso bamboo diversity was studied in four bamboo forests within a radius of 10 km. In each of those four bamboo forests, some leaves were collected from different culms at least 2 m apart. “JAPAN" is the same location as JAPAN in Fig. 3, so the bamboo grove is derived from a lineage first brought to Japan from China. The pedigrees of other bamboo groves are unknown. **b** Analysis using GRAS-Di revealed few loci were polymorphic among the samples. GATK 4.1.3.0 HaplotypeCaller was used for variant calling, with the –ERC option. The output was in vcf format. Filter 1 refers to the following: for all loci, removal of loci with DP < 10 according to GATK and samples with missing GQ; for SNPs, removal of loci with QD < 2.0 || FS > 60.0 || MQ < 40.0 || MQRankSum <  − 12.5 || ReadPosRankSum <  − 8.0; for InDels, removal of loci with QD < 2.0 || FS > 200.0 || ReadPosRankSum <  − 20.0. “Extraction of loci with newly generated HET” indicates selection only loci in which some samples were heterozygous and the rest were homozygous reference. Filter 2 refers to the following: removal of loci with [20 ≤ GQ < 90∧QUAL ≥ 160 or 90 ≤ GQ] or HET in 4, 8, or 12 samples from the same bamboo grove. See Additional file 6: Table S5 and Additional file 7: Table S6 for visual inspection and Sanger sequencing results. REF, homozygous reference; HET, heterozygous; ALT, homozygous alternative; HET in some, loci containing samples of the REF and HET but not the ALT; ALT in some, loci containing samples of the REF and ALT but not the HET; REF in all, REF in all tested samples; HET in all, HET in all tested samples; ALT in all, ALT in all tested samples; ND, no data

**Fig. 5:**
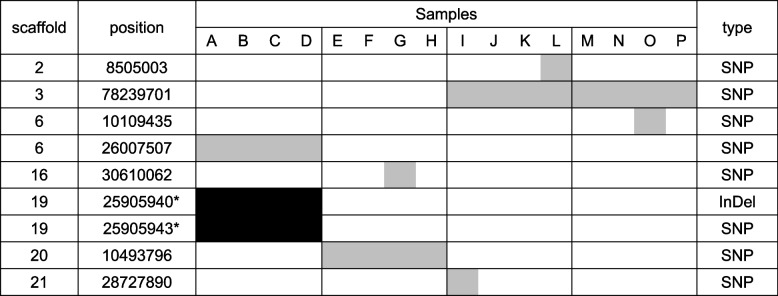
It appears that heterozygous loci arose in the bamboo grove and then spread within it. Gray background, heterozygous; black background, homozygous alternative. The sampling locations were JAPAN for ABCD, JAPAN2 for EFGH, JAPAN3 for IJKL, and JAPAN4 for MNOP. Sample D is the same as JAPAN sample analyzed by WGS. *A weak “homozygous reference sequence peak” was detected by Sanger sequencing: homozygous alternative samples indicated with the asterisk may be chimeric. Results of Sanger sequencing of the loci are shown in Additional file 7: Table S6. Homozygous alternative loci were unexpectedly detected. It is possible that heterozygous loci were generated by somatic mutation and spread within the bamboo grove, and that homozygous alternative loci were generated by a mechanism involving heterozygous loci

To understand the mechanisms underlying the genomic diversity of Moso bamboo, we sampled 16 specimens in the same and adjacent bamboo forests in Japan (Fig. 4a) and investigated the genomic diversity using GRAS-Di. The regions where all of the 16 samples analyzed by GRAS-Di had Depth≧10 were 6,080,028 bp in total. Also, when a cluster of consecutive bases with Depth≧10 in all 16 samples was counted as one region, 46,356 regions were amplified.　GRAS-Di analysis allows PCR amplification of a partial sequence of the genome without significant bias (Additional file 1: Fig. S13). Read length, volume, mapped read tables are summarized in Additional file 5: Table S4. In our analysis of the small area of Japan, we focused our analysis on heterozygous loci newly generated in Japan via somatic mutations (Fig. 4b), as we considered the trophically reproducing individuals are likely to have been distributed in the area. When genotyping using GRAS-Di data, we selected only loci in which some samples were heterozygous and the rest were homozygous reference. A vcf file format was used, and a non-stringent filter was applied to extract as many polymorphisms as possible. As variant calling is less accurate using heterozygous than homozygous alternatives, we then filtered the data with IGV v2.8.0 and visually inspected 507 loci (106 of which matched the GATK genotyping results) (Fig. 4b; Additional file 6: Table S5). As GRAS-Di uses PCR to amplify sequences, there is a possibility of amplification bias and errors (Additional file 1: Figs. S14, S15). Therefore, we also attempted to identify true polymorphisms by Sanger sequencing (Fig. 4b; Additional file 7: Table S6). The results revealed several loci that may have caused heterozygous mutations in the homozygous reference loci, which spread through the same bamboo grove or even became heterozygous among adjacent bamboo groves. Of the heterozygous and homozygous alternative loci in Fig. 5 identified by GRAS-Di analysis, polymorphisms were not found by WGS analysis, except for three polymorphisms in sample D. Sample D is identical to the JAPAN sample subjected to WGS analysis. These results again indicated that the Moso bamboo in Japan analyzed herein was likely derived from a single introduction from China. The observation of loci in which heterozygous mutations occurred within and among Moso bamboo groves indicated that heterozygous mutations led to diversity in Moso bamboo.

Homozygous alternative SNPs and InDels were widespread only within one bamboo grove (Fig. 5). Sanger sequencing of the homozygous alternative loci detected a weak reference sequence peak, suggesting that these loci may be chimeras (Additional file 1: Fig. S16). Surprisingly, homozygous alternative loci were detected by GRAS-Di analysis, even though we searched for heterozygous loci that may have arisen from somatic mutations in the homozygous reference loci. However, homozygous alternative loci were also detected by WGS (Fig. 1c; Additional file 1: Fig. S4), suggesting that homozygous alternative loci were generated from heterozygous loci by unknown mechanism(s) (Additional file 1: Fig. S5).

## Discussion

Moso bamboo is considered to have very little genomic diversity compared to *Arabidopsis thaliana* and Asian cultivated rice. Analyses of biallelic SNPs identified one SNP per 11 bp in 1,135 *A. thaliana* lines [28], one SNP per 16 bp in 3,010 rice lines [29], and one SNP per 351 bp in 427 Chinese Moso bamboo lines. Moreover, 94.82% of the SNPs were heterozygous in each individual [14]. In our analysis of 20 lineages of Moso bamboo, 0.37% (1 in 268 bp), of the approximately 1.1-Gb region analyzed may have comprised SNP/InDels or long deletions, including heterozygous loci (Fig. 1a). The small genomic diversity of Moso bamboo in our analysis of 20 whole-genome short-read data is consistent with previous report [14].

Molecular phylogenetic trees are commonly used to estimate phylogenetic relationships. However, there is currently no general evolutionary model that includes heterozygous loci. Evolutionary models such as JC69 [30] and K80 [31] use only homozygous loci; other methods should be considered for analysis of clonal plants, such as bamboo [10], which contain many heterozygous SNPs [14], because polymorphisms in the heterozygous state should be considered as a major factor in the generation of diversity in the absence of sexual reproduction [22]. The lack of an established phylogenetic analysis method using heterozygous loci, and the lower genotyping accuracy of variant calling using heterozygous compared to homozygous loci, made it challenging to estimate phylogenetic relationships in Moso bamboo. However, we were able to infer the origin of Japanese Moso bamboo, which could not be determined by PCA or clustering, by performing highly accurate genotyping using MQ as a filter, and through analysis of UpSet plots in terms of the heterozygosity among samples (Figs. 2b, c and 3; Additional file 1: Fig. S10a, b). Our proposed method is unique in that it does not require any numerical transformations, is suitable for estimating the phylogenetic relationship between the sample of interest and other samples, and is expected to have little effect on the results due to sample bias. Our method is applicable to clonal plants, such as bamboo. Various methods have been proposed to create phylogenetic trees using genotyping information including heterozygous loci, such as random haplotype sampling [32], the use of IUPAC codes [33], analysis of the identity by state distance [14], and haplotype-based phylogenetic analysis by calculating the distance from haplotypes including missing data and heterozygosity [34]. It is expected that phylogenetic analysis methods, including genotyping of heterozygous loci, will continue to be developed in the future.

In this work, most of the high-quality SNPs were heterozygous among all tested samples (Fig. 1b). We estimate that Moso bamboo plant bodies, rather than seeds, were transported from China to Japan on a ship about 300 years ago. These individuals may have had underground stems or underground stems, small aboveground culms, and leaves.

Phylogenetic relationships were explored by WGS analysis, which showed that the Chinese samples XA13 (Guangxi) and DA5 (Hunan) are distantly related to other Chinese samples, and to Japanese samples of Moso bamboo and kikko-chiku (Figs. 2c and 3; Additional file 1: Fig. S11). In a previous report, samples from Guangxi and Hunan were clustered into the same clade in the phylogenetic tree, and it was also inferred that Hunan may be the origin of Moso bamboo [14]. The results of our analyses were consistent with these conclusions. The relatively large number of 1,324 heterozygous mutations common to Japanese Moso bamboo and kikko-chiku (Fig. 2b, c) may indicate that WYS24 and the lineage of Moso bamboo introduced to Japan may have diverged at a relatively early stage.

Somatic mutations may have important consequences for plant evolution. As the Moso bamboo in Japan and China, and kikko-chiku in Japan, analyzed in this paper are considered to be a single clone, somatic mutations may play a particularly important role in the diversity and evolution of Moso bamboo. Somatic mutation rates vary among plant species and tissues [22, 35]. We analyzed genomes extracted from the leaves of 16 samples from four Japanese Moso bamboo groves using GRAS-Di, to investigate SNP/InDels with heterozygous polymorphisms in a region of about 6 Mbp. Only seven SNPs in heterozygous samples, and one SNP and one InDel in homozygous alternative samples, were found (Fig. 5). A previous study reported 9 × 10^−9^ mutations per base per year in rice tiller [22]. For Moso bamboo, the mutation rate was stated in a previous paper as 8.51 × 10^–8^ per generation [14]. In that paper, the generation time of Moso bamboo is considered to be 67 years, so the mutation rate of Moso bamboo is calculated to be 1.3 × 10^–9^ per base per year. In this study, If nine mutations occurred in a region of 6 Mbp after the introduction of Moso bamboo to Japan, 5 × 10^−9^ mutations per base per year would have occurred in the 300 years in the 4 bamboo groves since and 6 × 10^–10^ to 2 × 10^–9^ mutations per base per year per sample; Based on previous reports and the results of this study, Moso bamboo may thus have a mutation rate lower than rice.

While we could find no historical records of the introduction of kikko-chiku from China to Japan, our WGS analysis suggested that both of our two Japanese kikko-chiku samples were originated from a somatic mutation of Japanese Moso bamboo (Fig. 2b, c). We suggest that Japanese kikko-chiku may represent a dominant mutation caused by a novel heterozygous mutation that occurred in Moso bamboo in Japan, or a latent mutation (change from a heterozygous to homozygous alternative mutation). There are records of kikko-chiku occurring in Japan in 1951 [16] and before 1886 [36]. In addition, kikko-chiku existed in Japan before 1841 [37]. After the introduction of Moso bamboo from China to Japan, there have been a few cases of somatic mutations resulting in kikko-chiku in Japan, and it is thought that one or more of these lineages are still being cultivated in Japan today.

Unexpectedly, our GATK genotyping results obtained during GRAS-Di analysis were shown by Sanger sequencing to be inaccurate in many cases (Fig. 4b). It is more difficult to genotype heterozygous than homozygous loci. In addition, in GRAS-Di analysis, heterozygous loci may be incorrectly identified as homozygous reference or homozygous alternative due to bias associated with sample condition during PCR amplification (Additional file 1: Fig. S14), which may have occurred in our analysis (Additional file 1: Fig. S17). Furthermore, in GRAS-Di analysis, homozygous reference loci may be incorrectly identified as heterozygous loci due to errors in PCR amplification or an incomplete reference genome (i.e., missing sequences) (Additional file 1: Figs. S15, S18); again, this may have occurred in our analysis (Additional file 1: Fig. S19). In metagenome analysis, the reproducibility of amplicon sequencing is an issue [38, 39]. There is also controversy regarding the reproducibility of RAD-seq results [40]. Bias arising during PCR amplification could have a significant impact on the results of GRAS-Di analysis in some cases. When performing GBS, including GRAS-Di, bias during PCR amplification and incomplete reference genomes may affect the results if validation, such as Sanger sequencing or visual inspection using IGV, is not performed.

## Conclusions

In this study, we compared the genomes of the first introduced lineage of Moso bamboo in Japan, 2 lineages of Japanese kikko-chiku and 15 lineages of Moso bamboo from 15 regions in China. We inferred that all the lineages analyzed were clones derived from a single individual in a F2 or F3 genration after crossing between two *Phyllostachys* plants with a certain genetic distance. We also inferred that an individual closely related to the sample from Fujian, China, was introduced to Japan over the sea without seed reproduction and Japanese kikko-chiku we analyzed arose from a somatic mutation of Japanese Moso bamboo. Our method of inferring phylogenetic relationships by searching for common heterozygous loci among samples is unique and effective. Our method is applicable to clonal plants, such as bamboo. In addition, we collected 16 samples from four nearby bamboo forests in Japan and performed SNP and InDel analyses using GRAS-Di. The results suggested that a small number of somatic mutations would spread within and between bamboo groves.

## Methods

### Site description

Sampling of Japanese Moso bamboo was conducted at four sites in Shizuoka Prefecture, Japan. All samples were collected with the permission of the respective bamboo grove managers. We sampled JAPAN (35.1628, 138.8873) and JAPAN2 (35.1550, 138.8949) on December 29, 2019, JAPAN3 (35.1619, 138.9263) on January 3, 2020, and JAPAN4 (35.1592, 138.9389) on January 4, 2020. We collected four samples at each site. Multiple leaves were collected from one culm, which was considered as one sample. Samples were collected from different culms at least 2 m apart from each other and stored at − 20 °C thereafter. The four bamboo groves we sampled were all less than 10,000 square meters in area in a cultivated environment, and each bamboo grove is thought to have been spread by nutrient reproduction by individuals brought in by a single transplant. Therefore, each bamboo grove is considered to be derived from a single lineage. The Moso bamboo grove JAPAN was transplanted from Senganen in Kagoshima Prefecture, where Moso bamboo was first introduced to Japan. The origin of the Moso bamboo forests at JAPAN2, JAPAN3 and JAPAN4 are unknown. For kikko-chiku, the Kikko1 sample was taken at site (35.1619, 138.8856) and the Kikko2 sample was taken at site (35.1593, 138.8852), on October 6, 2020 in both cases. The origins of the Kikko1 and Kikko2 bamboo groves are unknown. We collected leaves on August 2, 2022 from germinated seeds of Moso bamboo from the Fuji Bamboo Garden that had flowered in 2021 [7].

### Extraction of total genomic DNA

Two or three leaves of Moso bamboo or kikko-chiku were placed in a mortar, to which liquid nitrogen was added, and the leaves were ground with a pestle. Then, total genomic DNA was extracted using the cetyltrimethylammonium bromide (CTAB) method [41] with minor modification.

### WGS and detection of the high-quality SNPs

For JAPAN and 8 Moso bamboo seedlings, libraries were prepared using TruSeq DNA PCR Free (350) (Illumina, San Diego, CA, USA). The sequencing data were collected using the NovaSeq 6000 instrument (Illumina) with 151-bp paired-ends, and converted into fastq data using the bcl2fastq2 tool without removing adapters. The data were then trimmed using Trimmomatic-0.39 [42] with PE-phred33 TOPHRED33 LEADING:3 TRAILING:3 SLIDINGWINDOW:4:15 MINLEN:36.

For kikko-chiku, a KAPA Hyper Prep Kit (for Illumina; Kapa Biosystems, Woburn, MA, USA) was used for library preparation. Data with 151-bp paired-ends were collected by NextSeq 500 (Illumina) and converted to fastq using the bcl2fastq2 tool, for adapter removal and fastq conversion.

We downloaded the raw Chinese Moso bamboo data from BioProject accession PRJNA755164 [14] and used the sra-toolkit/2.10.4 (https://github.com/ncbi/sra-tools) to convert the data to fastq format. Adapters were then detected in EARRINGS/1.0.0 [43] using the default settings. Adapter trimming was not performed for samples in which no adapters were detected by EARRINGS. Quality trimming was performed with trimmomatic-0.39 (PE -phred33 TOPHRED33 LEADING:3 TRAILING:3 SLIDINGWINDOW:4:15 MINLEN:36).

We downloaded the raw data used to create the reference genome of Moso bamboo [12] from SRA (ERR105036 to ERR105041 and SRR4120067). The data were then trimmed as described above.

The trimmed reads of each sample were mapped to the reference genome using bwa-mem2 (v2.2.1) [44] with the -M option. The reference genome was the chromosome-level Moso bamboo genome created using Hi-C [12]. The mapped reads were sorted and indexed using SAMtools 1.13 [45]. We then used Picard 2.26.5 [46] to check sequencing pair information and remove PCR duplicates. Each sample bam file was processed based on FixMateInformation SO = coordinate CREATE_INDEX = true VALIDATION_STRINGENCY = LENIENT, followed by MarkDuplicates CREATE_INDEX = true REMOVE_DUPLICATES = true ASSUME_SORTED = true VALIDATION_STRINGENCY = LENIENT, and then sorted and indexed with SAMtools 1.13.

We performed variant calling using GATK 4.2.3.0 and HaplotypeCaller with the -ERC BP_RESOLUTION –pcr-indel-model NONE options selected, created a gvcf file containing the information for each single nucleotide (genomic variant call format), and indexed it using IndexFeatureFile. Then, the gvcf files were combined into one file for all samples using the CombineGVCFs command in GATK 4.2.3.0, and genotyped using the GenotypeGVCFs command with the –include-non-variant-sites option. We then used snpshift (5.0) [47] to select only loci for which each sample satisfied the following: [DP > 4, and RGQ > 0], [DP > 4, and GQ > 0], or [DP = 0]. We then selected only loci for which DP < 100 for each sample, to remove loci with low reliability. Loci identified as homozygous alternative or other by the variant caller in the 125 bp paired-end or 250 bp paired-end short reads used when the reference genome was created [12] were excluded from the analyzed loci. "Other" indicates genotypes that are not homozygous reference, heterozygous, or homozygous alternative. We also excluded loci for which the genotype of all samples was the same as the reference genome. The above-described gvcf file was then converted into a tsv file using BCFtools 1.15.1. Only SNPs with MQ = 60 were extracted using R software 4.1.0 [48].

### GRAS-Di analysis

We used GRAS-Di analysis technology developed by Toyota Motor Corporation (Aichi, Japan) to analyze 16 samples of Moso bamboo from four sites in Shizuoka Prefecture, Japan (four samples per site). Sample D analyzed by GRAS-Di was identical to the JAPAN sample analyzed by WGS. The GRAS-Di library was constructed based on two sequential PCR analyses. In the first PCR, 63 different primers, consisting of the adapter sequence of Nextera (TAAGAGACAG) (Illumina) and 3-bp random sequences (except the primer with the 3′ end TGC) were used. The second PCR analysis included “multiplexing 8-base dual index” (Illumina) and P7/P5 adapter sequences. Data obtained with GRAS-Di were trimmed using Trimmomatic 0.38 (with ILLUMINACLIP:NexteraPE-PE.fa:2:30:10:2:true LEADING:3 TRAILING:3 SLIDINGWINDOW:4:15 MINLEN:36). Trimmed reads were mapped to the chromosome-level Moso bamboo genome [12] with BWA-0.7.17 (r1188) [49] using the “mem -M” option. Next, reads with MAPQ < 4 were excluded. Reads with no mate pair were also removed, along with soft- and hard-clipped reads. Then, bam files were sorted using SAMtools 1.8. Using GATK 4.1.3.0, the samples were processed using HaplotypeCaller with the options -ERC GVCF –pcr-indel-model CONSERVATIVE, indexed with IndexFeatureFile, combined with CombineGVCFs, genotyped with GenotypeGVCFs and filtered as follows: QD < 2.0 || FS > 60.0 || MQ < 40.0 || MQRankSum <  − 12.5 || ReadPosRankSum <  − 8.0' for SNPs; and QD < 2.0 || FS > 200.0 || ReadPosRankSum <  − 20.0' for InDels. BCFtools 1.9 was used to convert the vcf files to tsv files. Loci containing samples with DP < 10 by GATK output values and samples with missing GQ were removed using R 4.1.1. All subsequent analyses were performed using R 4.1.1.

### Sanger Sequencing and dCAPs

The primer sets used for Sanger sequencing or dCAPs [25] of loci, of which all tested samples were heterozygous in the WGS analysis were shown in Additional file 4: Table S3. For dCAPs analysis, electrophoresis was performed using DNA-500 with Multina, a microchip electrophoresis system for DNA analysis. The primer sets used for Sanger sequencing of loci, of which some tested samples were heterozygous in the GRAS-Di analysis are shown in Additional file 7: Table S6. We used BioEdit v7.2.5 [50] to check the Sanger sequencing data.

## Supplementary Information

**Additional file 1:** **Figure S1. **The culms of Moso bamboo and Kikko-chiku. Kikko-chiku is considered to be a variant with unusual culm shape from Moso bamboo. **Figure S2.** An overview of the WGS and GRAS-Di analysis. In the WGS analysis, only high-quality SNPs were selected for phylogenetic analysis, and in the GRAS-Di analysis, a lenient filter was used to prevent SNPInDels from being missed, followed by visual judgment using IGV, a genome viewer, and Sanger sequencing to confirm true polymorphisms.** Figure S3.** Distribution of MQ values for loci of candidate polymorphisms between samples. Among SNPs in the 4,103,158 loci candidate polymorphisms in Fig. 1a. There are 2,217,042 loci with MQ values, which are represented in the histogram. The area with yellow background is 529,072 loci with MQ=60. **Figure S4. ** Characteristics of the loci, including samples of ALT genotype. a There were 12,923 loci containing samples of ALT within high quality SNPs. "Removal of loci for ALT sample only" indicates the removal of loci for which all 18 data are ALT, excluding the two data from the reference genome creation. b Most loci that included samples of ALT included samples of HET and did not include samples of REF. “ALT” indicates that there were only ALT samples. “ALT, HET” indicates that there were samples of ALT and HET, but no samples of REF. “ALT, REF” indicates that there was a sample of ALT and a sample of REF, but no sample of HET. “ALT, HET, REF” indicates that there were samples of ALT, samples of HET, and samples of REF. **c** Most of the 12,923 loci containing samples of ALT, only one sample was ALT and the remaining 17 samples were HET. ALT may have arisen from HET by homologous recombination (HR) repair or other means without sexual reproduction. These analyses were performed on 18 data, excluding 2 data used to create the reference genome. REF, homozygous reference; HET, heterozygous; ALT, homozygous alternative. **Figure S5. **A possible model in which ALT occurs in Moso bamboo without sexual reproduction. HET, heterozygous; ALT, homozygous alternative. **Figure S6. **Distribution of high-quality SNPs a Distribution of high-quality SNPs. The distribution of high-quality SNPs in the 414,952 loci was somewhat skewed, but the 97,869 loci, excluding loci for which all samples were heterozygous or all samples were homozygous alternatives, were evenly distributed throughout the genome. b Histogram of the position of the high-quality SNPs on scaffold 3 and the GenMap analysis of the scaffold 3. GenMap analysis was conducted with the parameter –K 30 –E2. The output wig file was visualized using IGV 2.11.1. The histogram was created using the R4.1.0 function hist. The width of the bin is 1,000,000 bp. **Figure S7.** Heterozygous loci in the parent is reduced in progenies, according to Mendel’s law of segregation. When mating occurs, HET loci in Parent 1 are expected to be REF or ALT in progenies in certain ratio. REF, homozygous reference; HET, heterozygous; ALT, homozygous alternative. **Figure S8.** Investigation of genotype in 8 individual seedlings. a In 1954 in Kyoto, Japan, a Moso bamboo flowered. The number of seeds obtained is unknown but the seedlings were cultivated and grown at the Fuji Bamboo Garden and flowers bloomed in 2021. 8 seedlings obtained were cultivated and WGS analysis was performed. According to Mendel‘s law of segregation, HET loci should segregate to REF: HET: ALT= 3:2:3 at 8 seedlings. b 11 whole-genome short-read data were used for WGS analysis. Moso bamboo thought to have been first introduced to Japan, the 8 seedlings and 125- and 250-bp short reads used when the reference genome was created were analyzed. c Genotypes for each sample in 549,916 high-quality SNPs. d Extraction of loci, where the “JAPAN” is HET was performed. Genotypes for each sample in the 520,806 SNPs is shown in the bar graph. e Among the 520,806 SNPs extracted in d, the distribution of SNPs in scaffold 3, which is a chromosome-level scaffold, was investigated in each sample. The bin width is 500,000 bp. The hist function of R4.1.0 was used to draw the figure. Scaffold 3: 62,305,000-62,307,000 area shown enlarged. In this region, there is one SNP in each bin. f A note about the histograms in e. Moso bamboo as well as most of eukaryotes have 2 haplotypes. The Moso bamboo reference genome [12] was constructed using short-read sequencing data. Therefore, it is likely that the reference genome has fragmented haplotypes. In seedlings, there should be both homozygous fixed loci and heterozygous loci. However, in the homozygous-fixed region, the heterozygous reference loci and the homozygous alternative loci should be randomly distributed when compared with the reference genome. In the histogram, REF, HET, and ALT were aggregated and stacked vertically within the same bin. REF, homozygous reference; HET, heterozygous; ALT, homozygous alternative. **Figure S9.** Summary of genotypes for each sample a Genotypes for each sample of 414,952 high-quality SNPs shown in Fig. 1a. b The genotype of each sample of loci where the "JAPAN" sample is HET. REF, homozygous reference; HET, heterozygous; ALT, homozygous alternative. **c **Distribution in each sample of 21,969 loci of scaffold3 out of 350,393 loci shown in b. The bin width is 500,000 bp. **Figure S10. **Clustering and PCA could not deduce the origin of Japanese Moso bamboo. The 9,104 loci used in Fig. 2a were analyzed. Genotype was converted to homozygous reference=0, heterozygous=0.5, homozygous alternative=1. a The clustering was performed using pvclust of R package. The analysis condition is Euclidean distance, Ward.D2 method, nboot=1000. b PCA was performed using the R function prcomp. **Figure S11.** Analysis of the origin of Japanese Moso bamboo by analysis of heterozygous SNPs common among samples. The selected 4,750 SNPs shown in Fig. 2a were analyzed using UpSet plots. UpSet plots are alternative to the Venn Diagram used to deal with more than 3 sets. These figures were created using an R package called ComplexUpset. Analysis was performed under the condition of “sort_intersections_by = c (‘degree’,‘cardinality’)”. For sample combinations other than 15 and 16, all sample combinations are shown. For sample combinations with 15 heterozygous samples, only sample combinations with the minimum number of loci of 2 or more than 2 are shown. For sample combinations with 16 heterozygous samples, only sample combinations with the minimum number of loci of 10 or more than 10 are shown. HET, heterozygous. **Figure S12. **Clustering analysis without 2 kikko-chiku samples. a With the exclusion of the 2 samples of Japanese Kikko-chiku, loci with no polymorphism among samples or one sample having a different genotype from the others were excluded. b The 7,376 loci were analyzed. Genotype was converted to homozygous reference=0, heterozygous=0.5, homozygous alternative=1. The clustering was performed using pvclust of R package. The analysis condition is Euclidean distance, Ward.D2 method, nboot=1,000. **Figure S13. **Partial sequences of the Moso bamboo genome were evenly amplified from the genome using GRAS-Di. We plotted the tip loci of each amplified region with a depth of 10 or more for all 16 samples. a All scaffolds. b Scaffold1. **Figure S14.** A model in which heterozygous loci are falsely detected as different genotypes in GRAS-Di analysis. Red letters indicate incorrect genotyping. REF, homozygous reference; HET, heterozygous; ALT, homozygous alternative. **Figure S15. **A model in which REF loci are falsely detected as HET in the GRAS-Di analysis. This model is a model in which PCR error causes incorrect genotyping. Red letters indicate incorrect genotyping. REF, homozygous reference; HET, heterozygous.** Figure S16. **Among the homozygous alternative loci of Moso bamboo, there were loci that may be chimeras. The loci in this figure, which were determined to be heterozygous by GRAS-Di analysis, were checked by Sanger sequencing and were thought to be homozygous alternative, but could be chimeras. In the 2 homozygous alternative loci, weak reference sequence signals were also visible, suggesting chimeras. **Figure S17.** In the GRAS-Di analysis, incorrect genotyping occasionally occurred due to bias during PCR. This figure shows as an example of the model shown in Additional file1: Fig. S14. In the GRAS-Di analysis, genotypes 2551827 and 25518531 of Scaffold7 in Sample D were determined inaccurately due to bias during PCR. The upper half figure shows the results of GRAS-Di amplified sequences with IGV. The lower half of the figure shows the result of Sangar sequencing. Scaffold7:25518527 in Sample D was ALT and scaffold7:25518531 was REF in the GRAS-Di analysis, but both loci were heterozygous in the Sangar sequencing. Red letters indicate incorrect genotyping. REF, homozygous reference; HET, heterozygous; ALT; homozygous alternative. **Figure S18. **A model in which REF is falsely detected as HET in the GRAS-Di analysis. a Homozygous reference loci is correctly determined to be a homozygous reference if the reference genome is complete. b This figure shows the case where some sequences of the genome are missing from the reference genome. If the missing sequence in the reference genome is amplified or not due to bias during PCR, the homozygous reference loci may be correctly determined to be homozygous reference, but may also be determined to be HET due to incorrect mapping. Red letters indicate incorrect genotyping. REF, homozygous reference; HET, heterozygous. **Figure S19.** In the GRAS-Di analysis, homozygous reference loci were occasionally incorrectly determined to be heterozygous loci. In GRAS-Di analysis, genotypes of 13981116, 13981148, and 13981151 of Scaffold5 in Sample B were all heterozygous, but those loci were all homozygous reference in Sanger sequencing. In the GRAS-Di analysis, 13 samples of 13981116 from Scaffold 5 and 14 samples of 13981148 and 13981151 from Scaffold 5 were incorrectly determined as heterozygous. In the PCR error model in Additional file 1: Fig. S15, it is unlikely that the same error would occur in the same loci of a large number of samples. Probably, these errors were caused by "incomplete reference genome and bias during PCR", which corresponds to Additional file 1: Fig. S18b. REF, homozygous reference; HET, heterozygous.

**Additional file2:** **Table S1.** Visual inspection of 150 of the 319,431 loci for which all samples were heterozygous.

**Additional file3:** **Table S2.** Sangar sequencing and dCAPs results for loci for which all samples tested in the WGS analysis were heterozygous.

**Additional file4:** **Table S3.** Primers and restriction enzymes used for Sangar sequencing and dCAPs.

**Additional file5:** **Table S4.** Read length, volume, and mapped reads for GRAS-Di analysis.

**Additional file 6:** **Table S5. **GATK genotyping results using GRAS-Di data were visually inspected using IGV.

**Additional file 7:** **Table S6.** Inspection of polymorphisms detected by GRAS-Di analysis with Sanger sequencing.

## Data Availability

The datasets generated and/or analyzed during the current study are available in the DDBJ repository, PRJDB14590, PRJDB14591, PRJDB14592 and PRJDB15604.

## Abbreviations

GATK: Genome Analysis Toolkit
GBS: Genotyping by sequencing
GRAS-Di: Genotyping by Random Amplicon Sequencing-Direct
IGV: Integrative Genomics Viewer
InDel: Insertion/deletion
PCA: Principal component analysis
SNP: Single nucleotide polymorphisms
WGS: Whole-genome resequencing
