# Supplementary material for: High genome heterozygosity revealed vegetative propagation over the sea in Moso bamboo
Source: BMC Genomics. 2023 Jun 24;24:348. doi: 10.1186/s12864-023-09428-9 (PMC10290394; doi:10.1186/s12864-023-09428-9)
Supplement: Supplementary file 1 — Additional file 1: Figure S1. The culms of Moso bamboo and Kikko-chiku. Kikko-chiku is considered to be a variant with unusual culm shape from Moso bamboo. Figure S2. An overview of the WGS and GRAS-Di analysis. In the WGS analysis, only high-quality SNPs were selected for phylogenetic analysis, and in the GRAS-Di analysis, a lenient filter was used to prevent SNPInDels from being missed, followed by visual judgment using IGV, a genome viewer, and Sanger sequencing to confirm true polymorphisms. Figure S3. Distribution of MQ values for loci of candidate polymorphisms between samples. Among SNPs in the 4,103,158 loci candidate polymorphisms in Fig. 1a. There are 2,217,042 loci with MQ values, which are represented in the histogram. The area with yellow background is 529,072 loci with MQ=60. Figure S4. Characteristics of the loci, including samples of ALT genotype. a There were 12,923 loci containing samples of ALT within high quality SNPs. "Removal of loci for ALT sample only" indicates the removal of loci for which all 18 data are ALT, excluding the two data from the reference genome creation. b Most loci that included samples of ALT included samples of HET and did not include samples of REF. “ALT” indicates that there were only ALT samples. “ALT, HET” indicates that there were samples of ALT and HET, but no samples of REF. “ALT, REF” indicates that there was a sample of ALT and a sample of REF, but no sample of HET. “ALT, HET, REF” indicates that there were samples of ALT, samples of HET, and samples of REF. c Most of the 12,923 loci containing samples of ALT, only one sample was ALT and the remaining 17 samples were HET. ALT may have arisen from HET by homologous recombination (HR) repair or other means without sexual reproduction. These analyses were performed on 18 data, excluding 2 data used to create the reference genome. REF, homozygous reference; HET, heterozygous; ALT, homozygous alternative. Figure S5. A possible model in which ALT occurs in Moso bamboo wi [file 12864_2023_9428_MOESM1_ESM.docx]

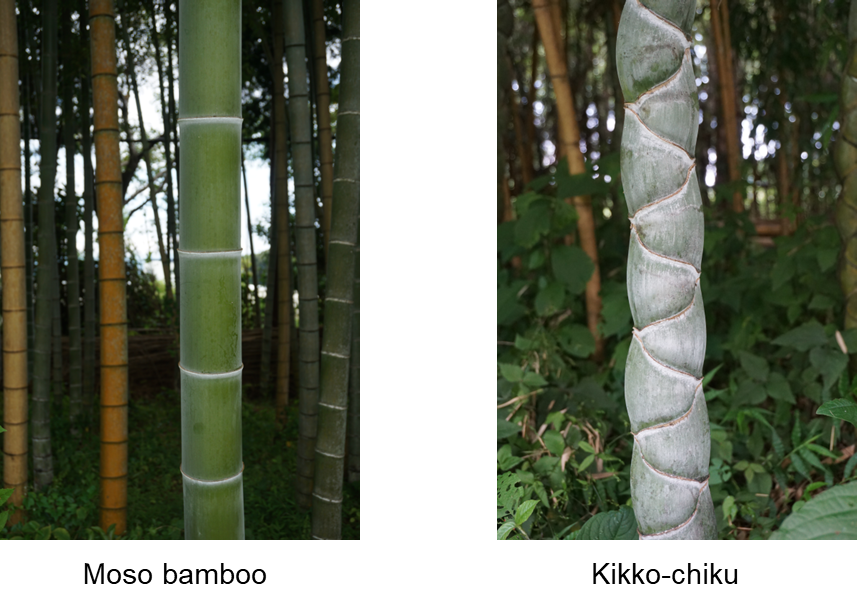
**Figure S1.** The culms of Moso bamboo and Kikko-chiku. Kikko-chiku is considered to be a variant with unusual culm shape from Moso bamboo.

**
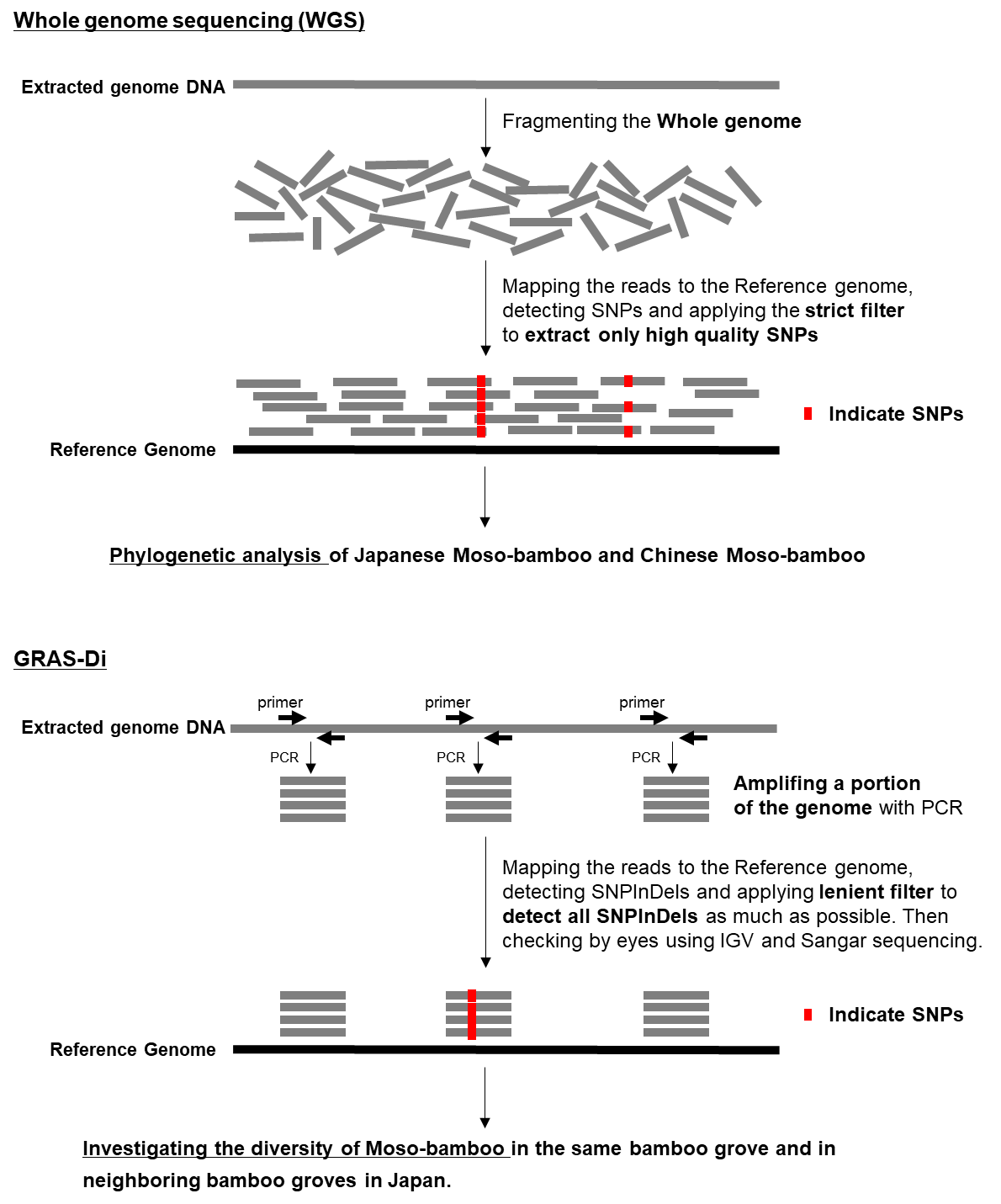
Figure S2.** An overview of the WGS and GRAS-Di analysis. In the WGS analysis, only high-quality SNPs were selected for phylogenetic analysis, and in the GRAS-Di analysis, a lenient filter was used to prevent SNPInDels from being missed, followed by visual judgment using IGV, a genome viewer, and Sanger sequencing to confirm true

polymorphisms.


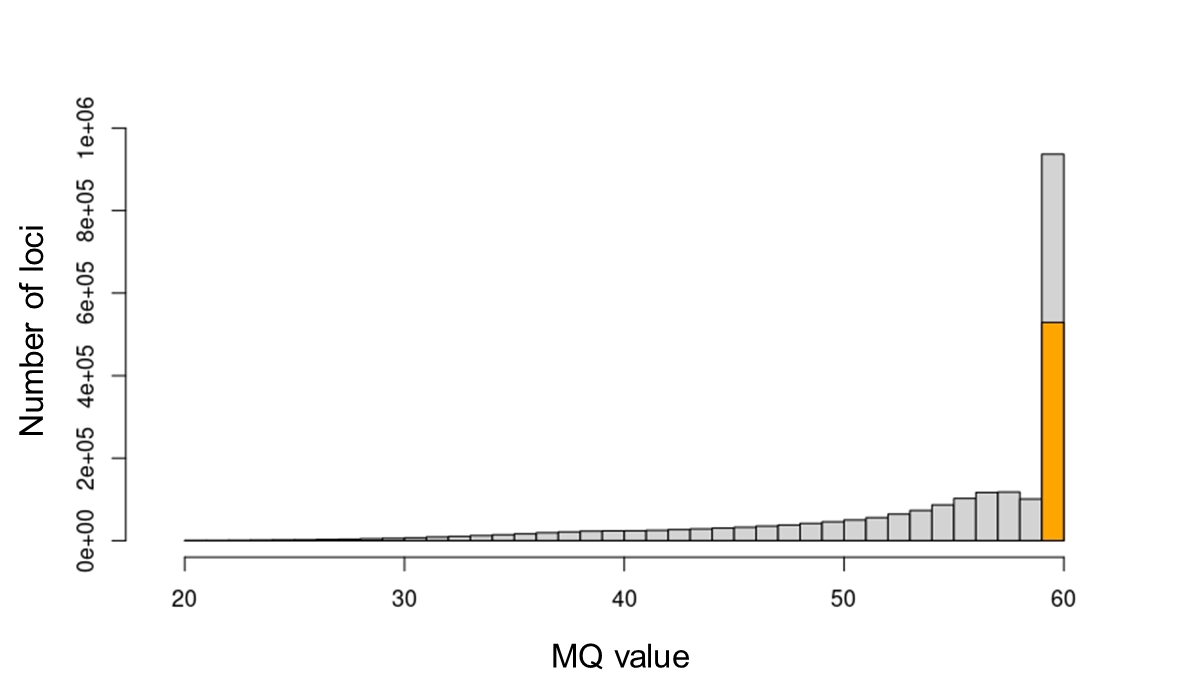
**Figure S3.** Distribution of MQ values for loci of candidate polymorphisms between samples. Among SNPs in the 4,103,158 loci candidate polymorphisms in Figure 1a. There are 2,217,042 loci with MQ values, which are represented in the histogram. The area with yellow background is 529,072 loci with MQ=60.


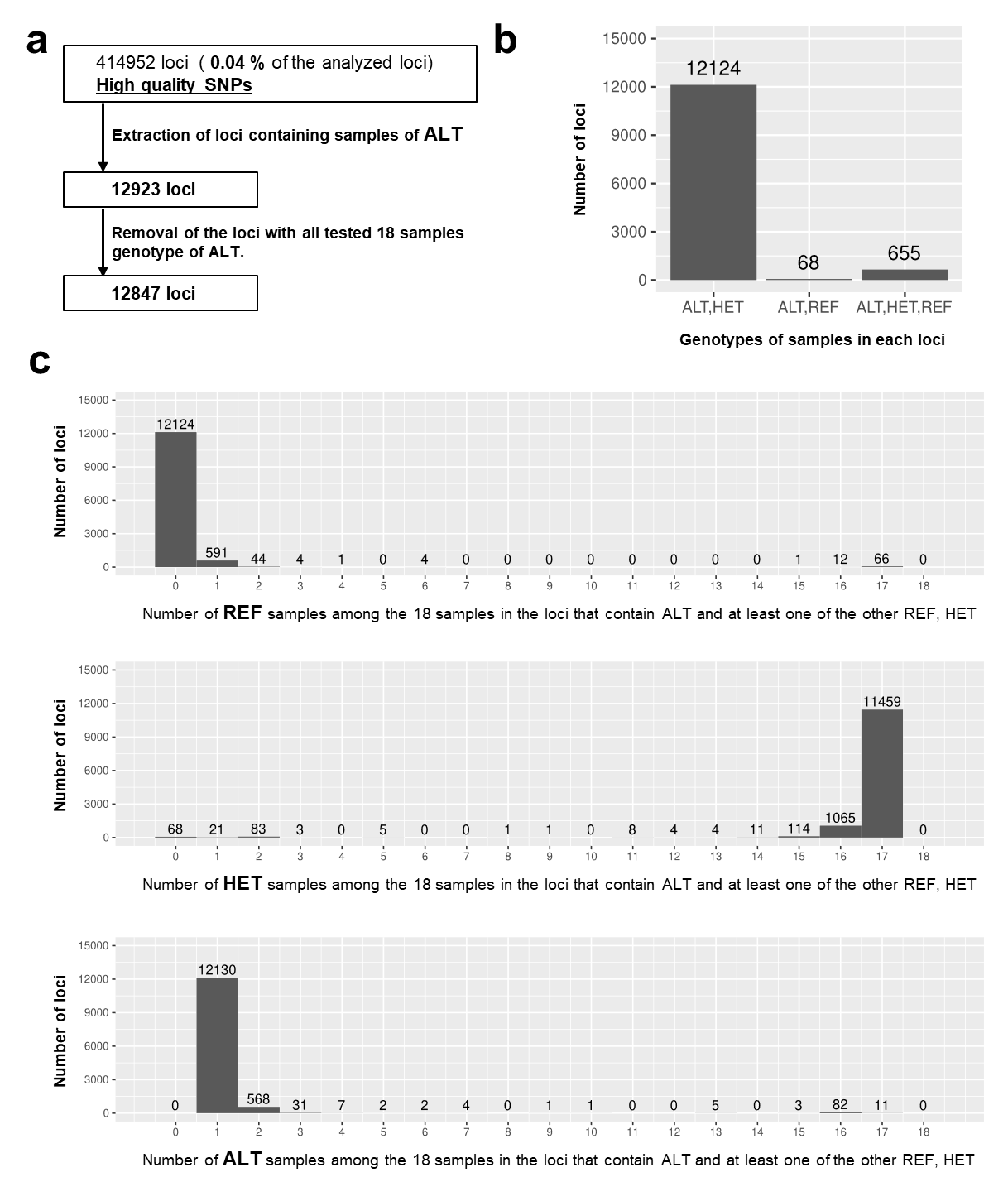


**Figure S4.**  Characteristics of the loci, including samples of ALT genotype. **a** There were 12,923 loci containing samples of ALT within high quality SNPs. "Removal of loci for ALT sample only" indicates the removal of loci for which all 18 data are ALT, excluding the two data from the reference genome creation. **b** Most loci that included samples of ALT included samples of HET and did not include samples of REF. “ALT” indicates that there were only ALT samples. “ALT, HET” indicates that there were samples of ALT and HET, but no samples of REF. “ALT, REF” indicates that there was a sample of ALT and a sample of REF, but no sample of HET. “ALT, HET, REF” indicates that there were samples of ALT, samples of HET, and samples of REF. **c** Most of the 12,923 loci containing samples of ALT, only one sample was ALT and the remaining 17 samples were HET. ALT may have arisen from HET by homologous recombination (HR) repair or other means without sexual reproduction. These analyses were performed on 18 data, excluding 2 data used to create the reference genome. REF, homozygous reference; HET, heterozygous; ALT, homozygous alternative.


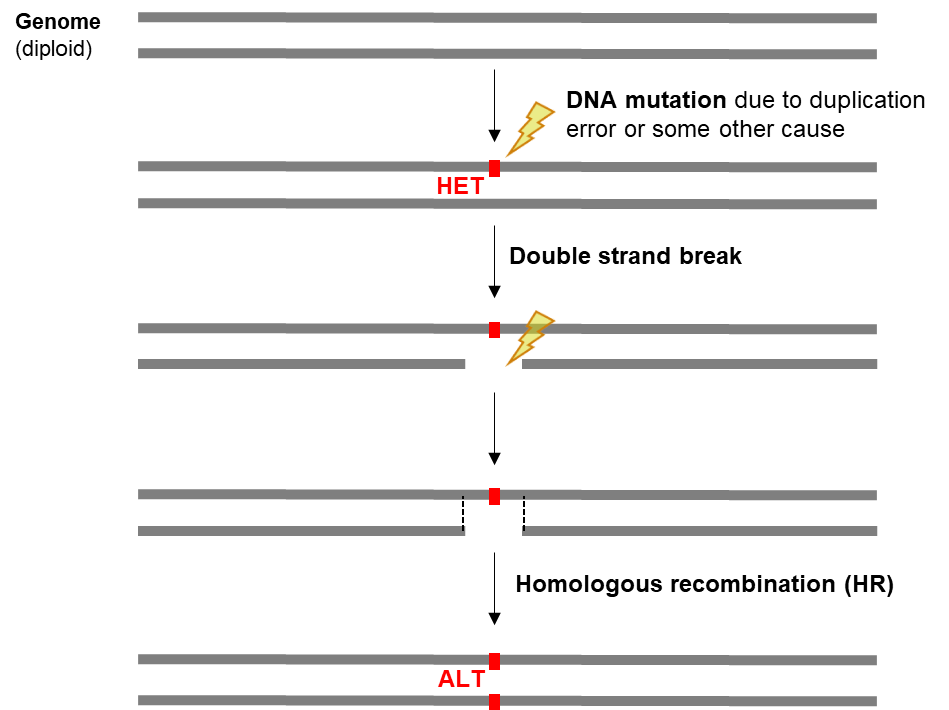


**Figure S5.** A possible model in which ALT occurs in Moso bamboo without sexual reproduction. HET, heterozygous; ALT, homozygous alternative.


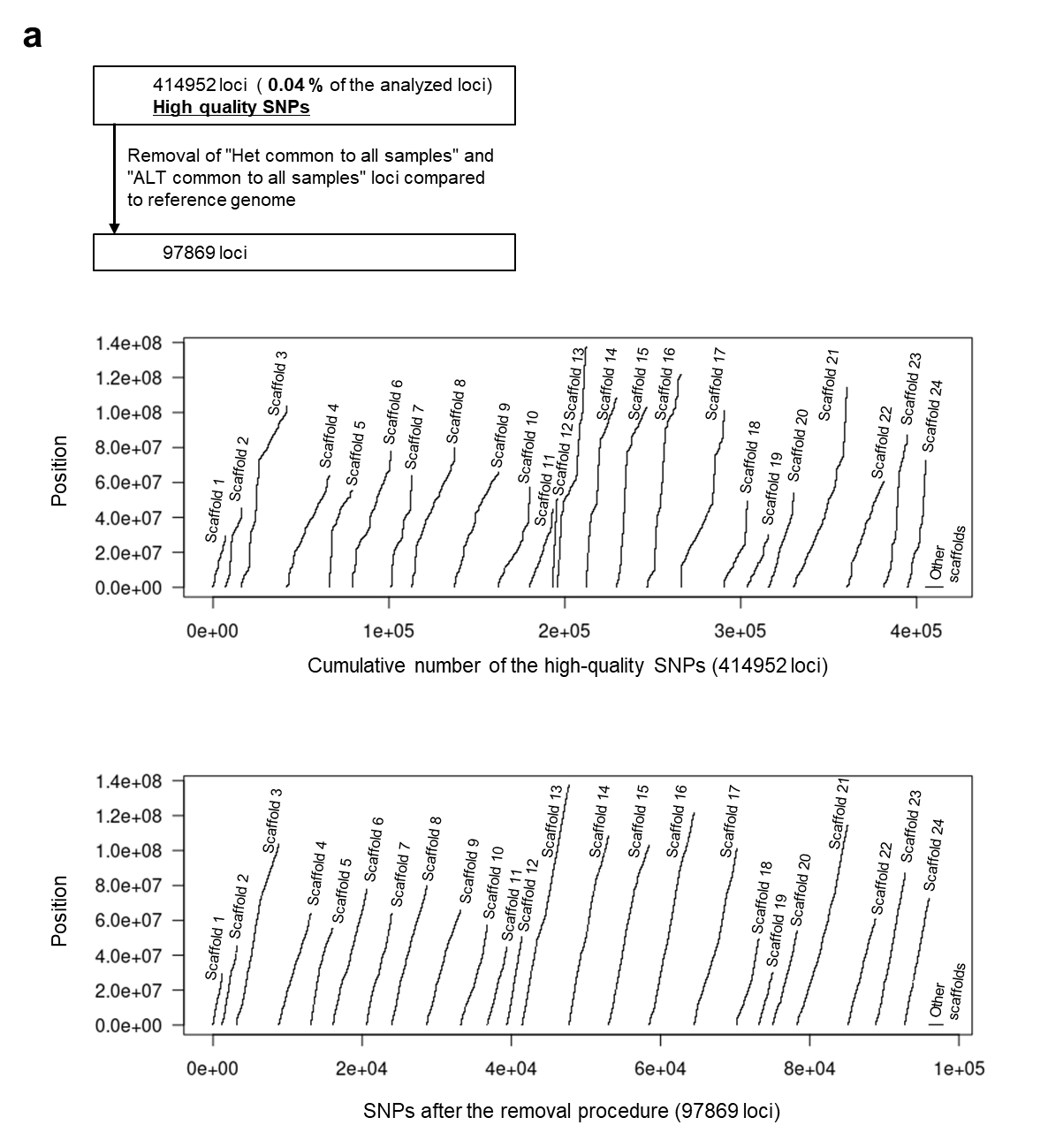


**
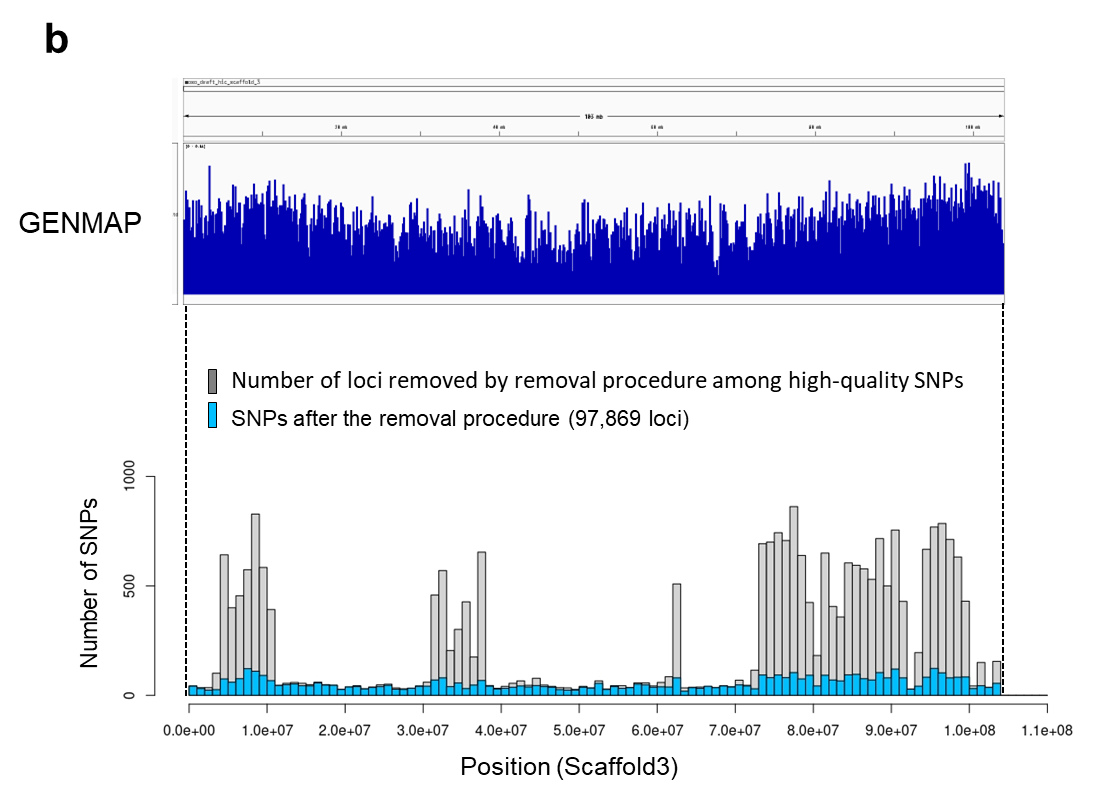
**

**Figure S6.** Distribution of high-quality SNPs

**a** Distribution of high-quality SNPs. The distribution of high-quality SNPs in the 414,952 loci was somewhat skewed, but the 97,869 loci, excluding loci for which all samples were heterozygous or all samples were homozygous alternatives, were evenly distributed throughout the genome. **b** Histogram of the position of the high-quality SNPs on scaffold 3 and the GenMap analysis of the scaffold 3. GenMap analysis was conducted with the parameter –K 30 –E2. The output wig file was visualized using IGV 2.11.1. The histogram was created using the R4.1.0 function hist. The width of the bin is 1,000,000 bp.

**
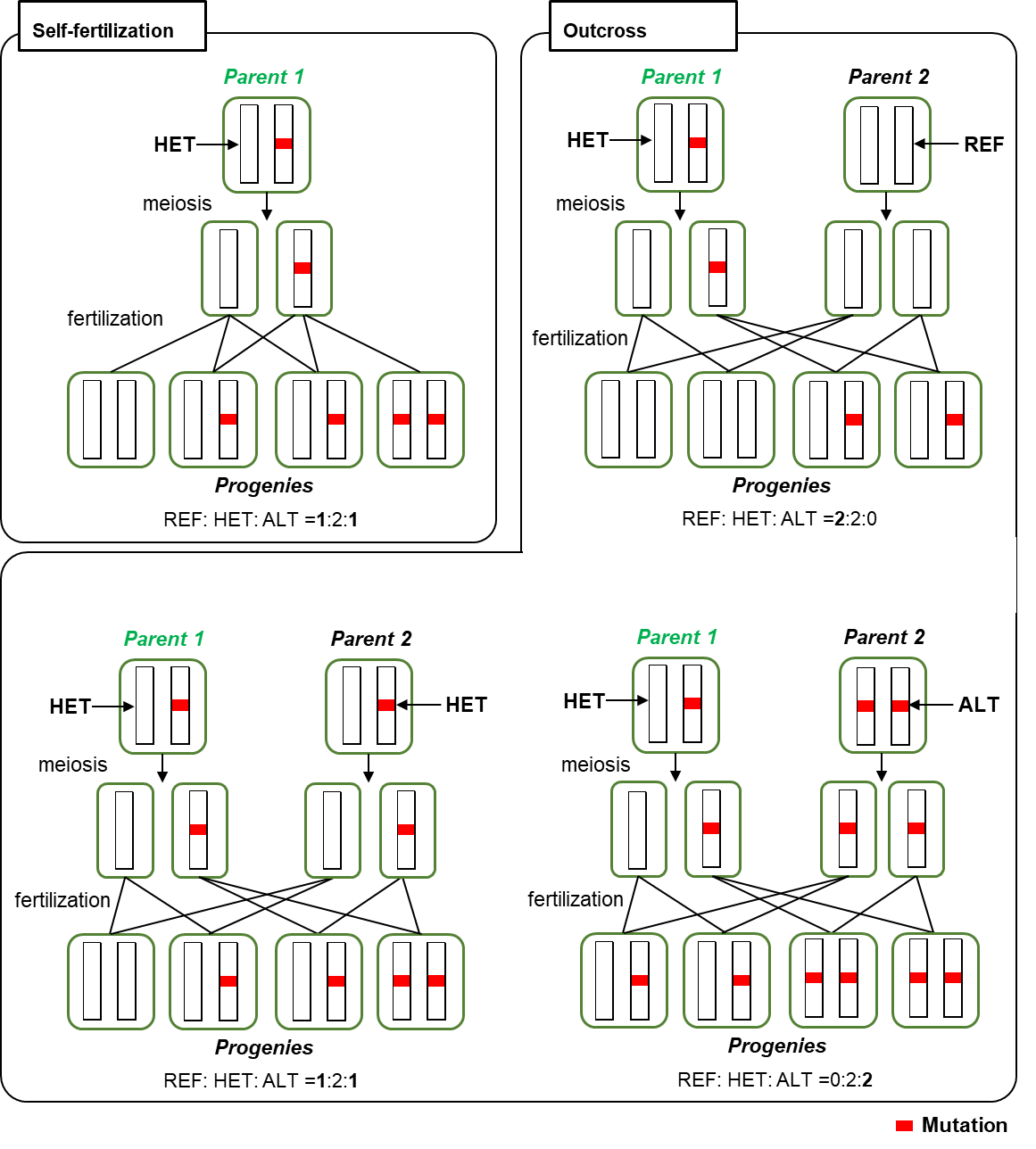
**

**Figure S7.** Heterozygous loci in the parent is reduced in progenies, according to Mendel’s law of segregation. When mating occurs, HET loci in Parent 1 are expected to be REF or ALT in progenies in certain ratio. REF, homozygous reference; HET, heterozygous; ALT, homozygous alternative.


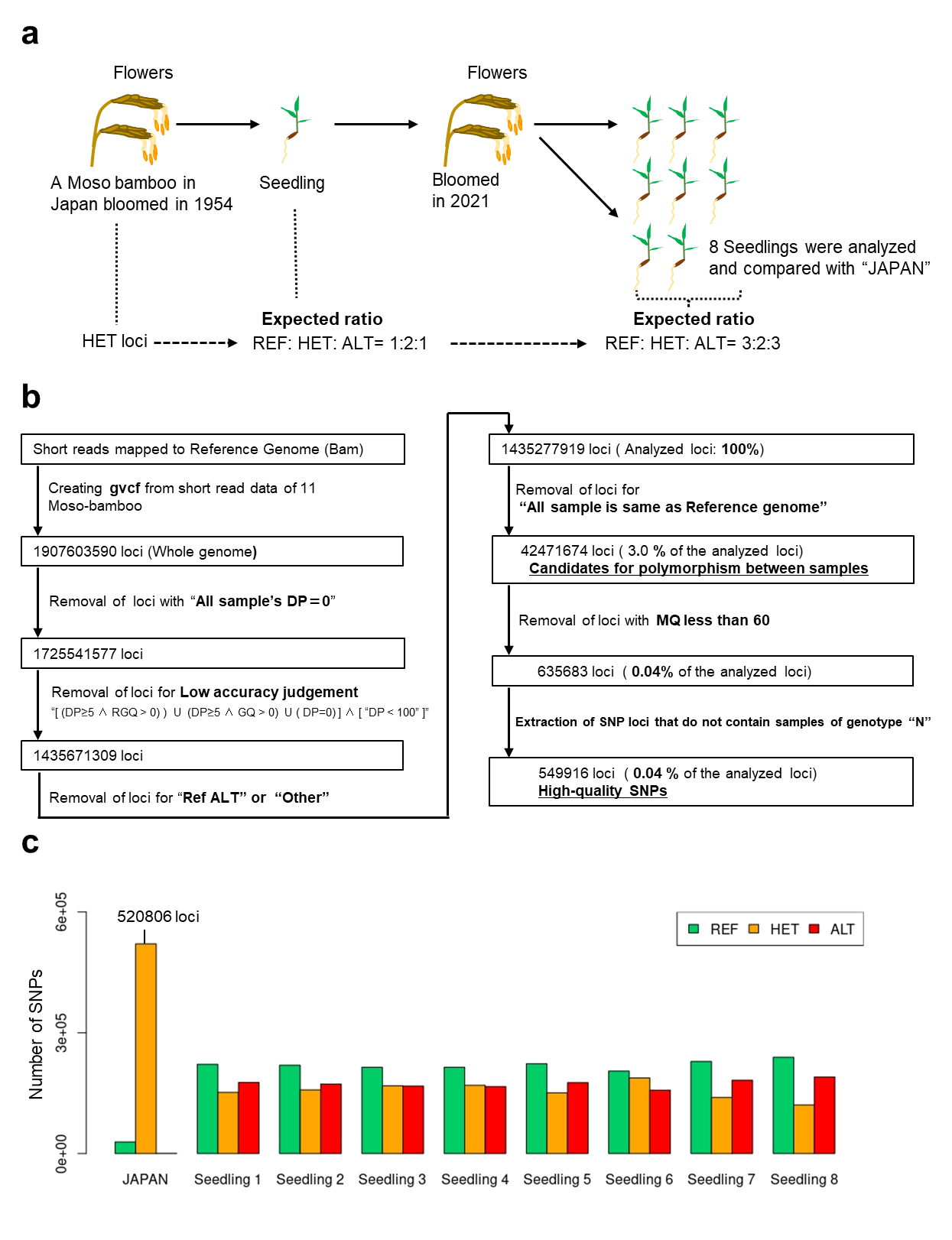


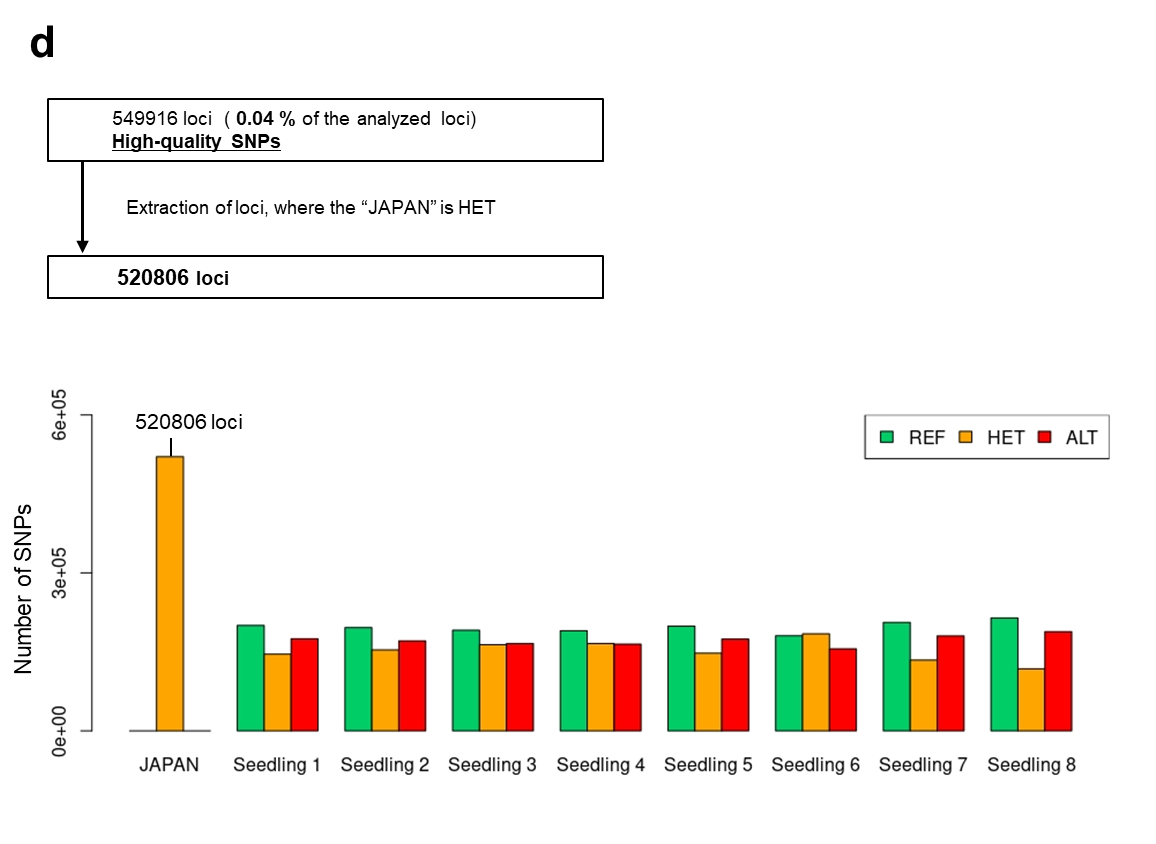


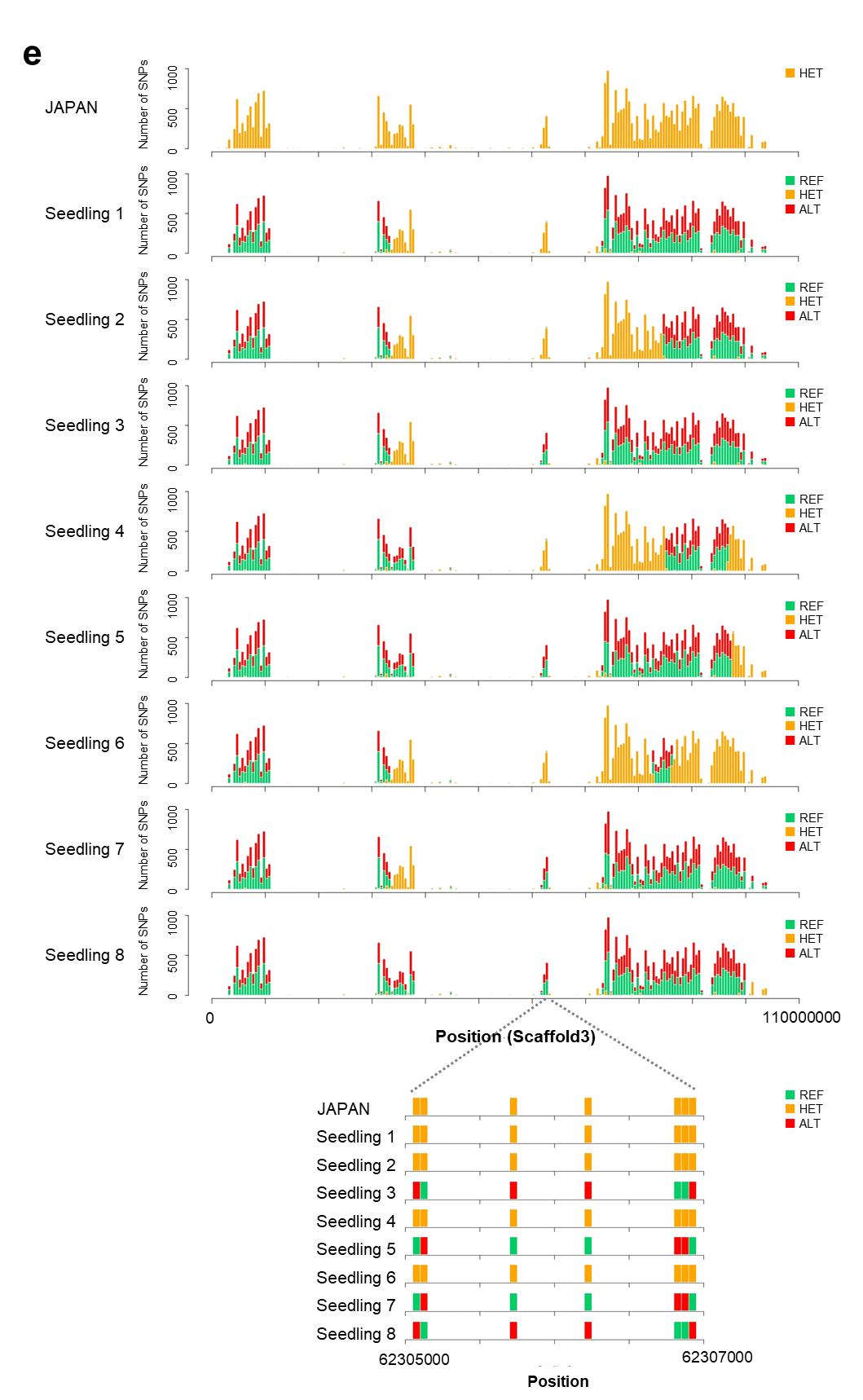


**
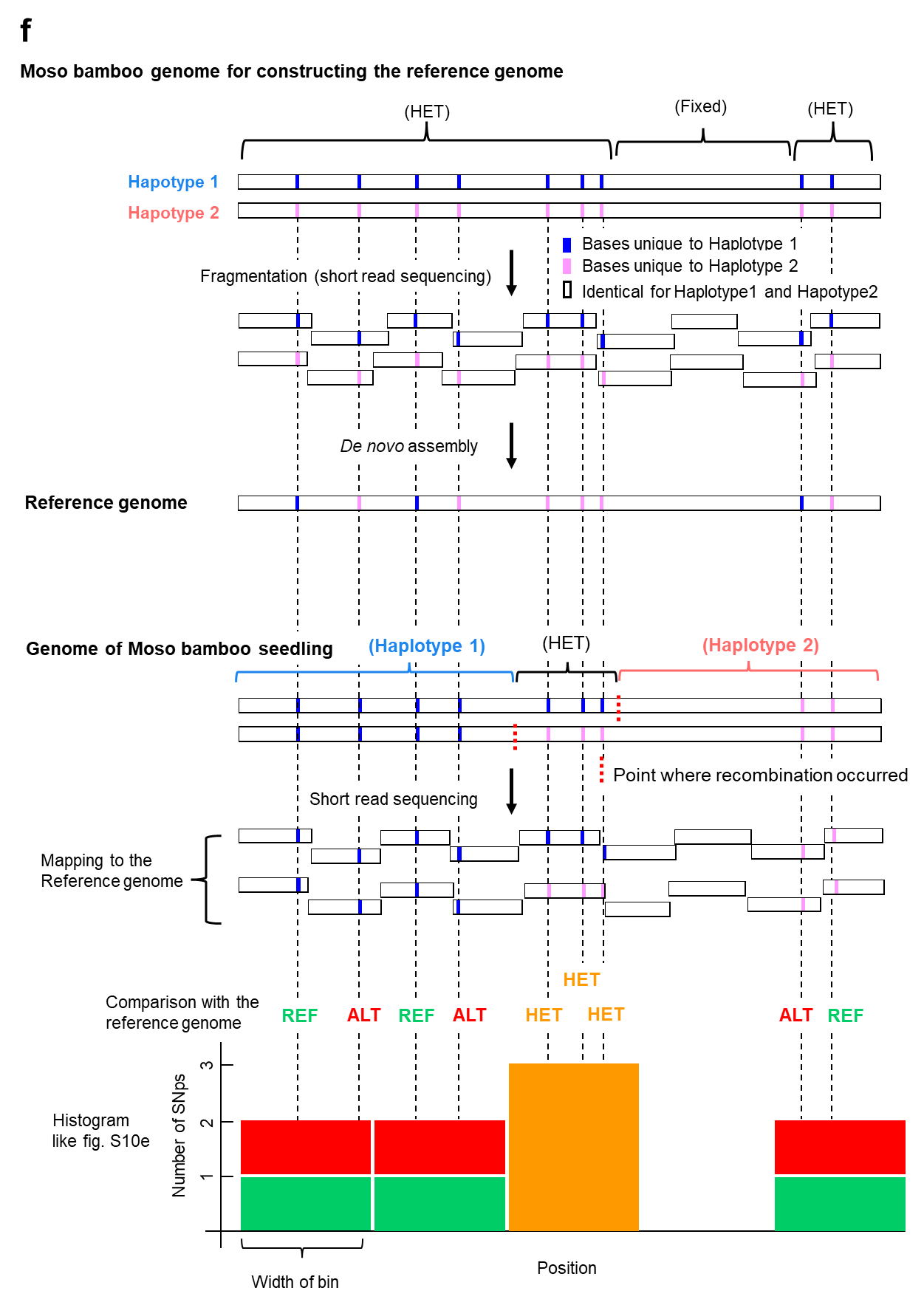
**

**Figure S8.** Investigation of genotype in 8 individual seedlings.

**a** In 1954 in Kyoto, Japan, a Moso bamboo flowered. The number of seeds obtained is unknown but the seedlings were cultivated and grown at the Fuji Bamboo Garden and flowers bloomed in 2021. 8 seedlings obtained were cultivated and WGS analysis was performed. According to Mendel‘s law of segregation, HET loci should segregate to REF: HET: ALT= 3:2:3 at 8 seedlings. **b** 11 whole-genome short-read data were used for WGS analysis. Moso bamboo thought to have been first introduced to Japan, the 8 seedlings and 125- and 250-bp short reads used when the reference genome was created were analyzed. **c** Genotypes for each sample in 549,916 high-quality SNPs. **d** Extraction of loci, where the “JAPAN” is HET was performed. Genotypes for each sample in the 520,806 SNPs is shown in the bar graph. **e** Among the 520,806 SNPs extracted in d, the distribution of SNPs in scaffold 3, which is a chromosome-level scaffold, was investigated in each sample. The bin width is 500,000 bp. The hist function of R4.1.0 was used to draw the figure. Scaffold 3: 62,305,000-62,307,000 area shown enlarged. In this region, there is one SNP in each bin. **f** A note about the histograms in e. Moso bamboo as well as most of eukaryotes have 2 haplotypes. The Moso bamboo reference genome [12] was constructed using short-read sequencing data. Therefore, it is likely that the reference genome has fragmented haplotypes. In seedlings, there should be both homozygous fixed loci and heterozygous loci. However, in the homozygous-fixed region, the heterozygous reference loci and the homozygous alternative loci should be randomly distributed when compared with the reference genome. In the histogram, REF, HET, and ALT were aggregated and stacked vertically within the same bin. REF, homozygous reference; HET, heterozygous; ALT, homozygous alternative.


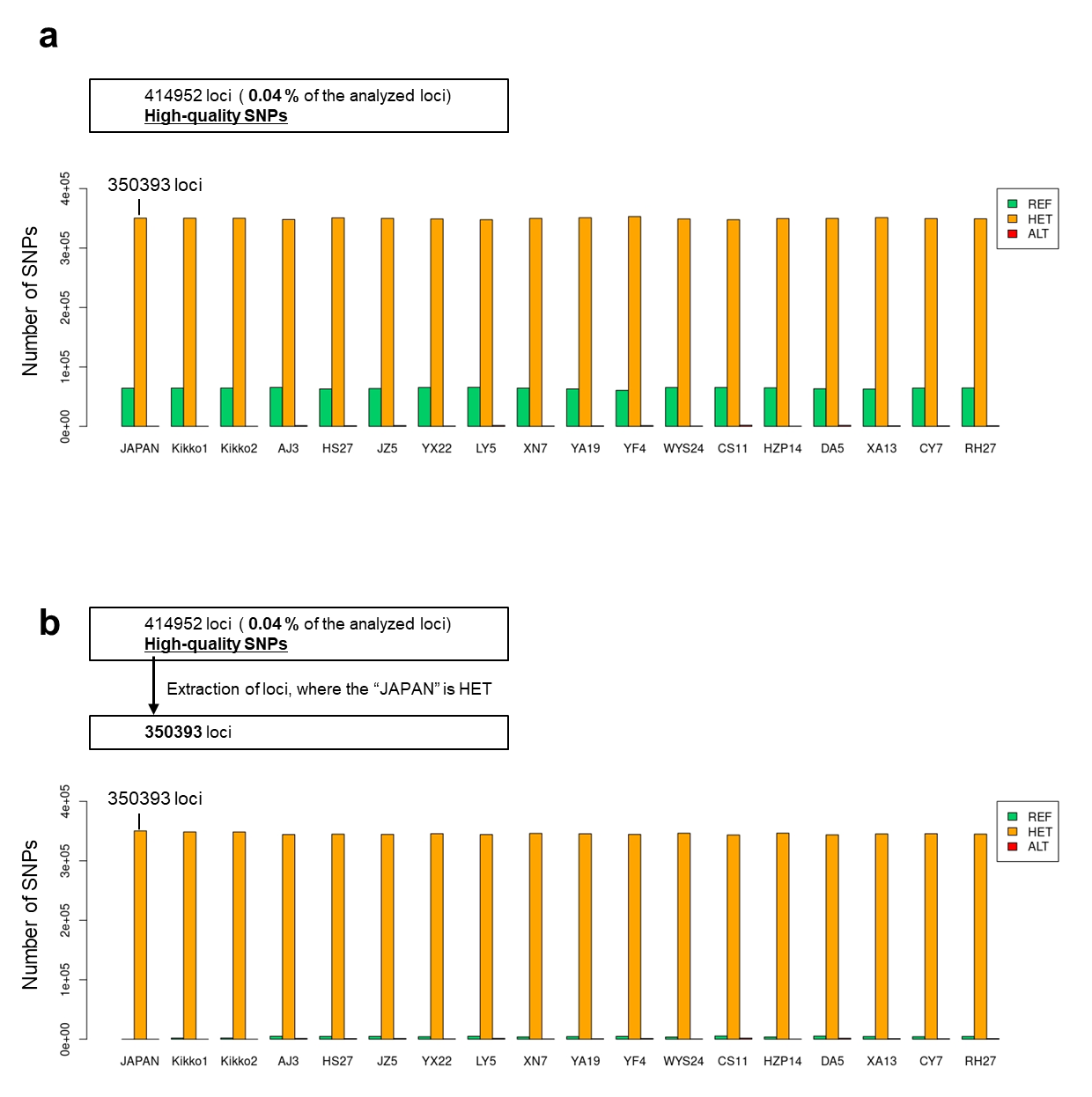


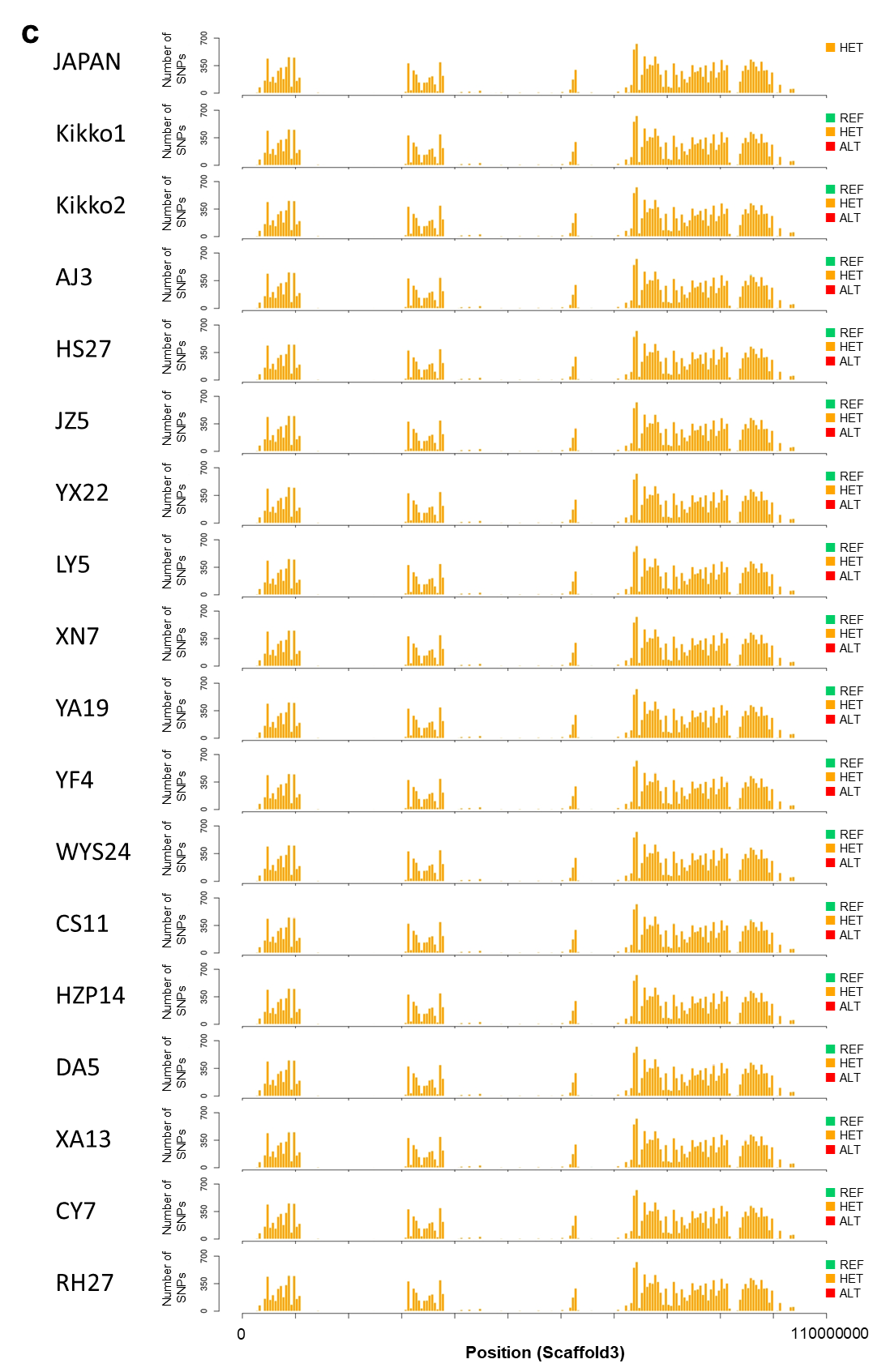


**Figure S9.** Summary of genotypes for each sample

**a** Genotypes for each sample of 414,952 high-quality SNPs shown in Fig. 1a. **b** The genotype of each sample of loci where the "JAPAN" sample is HET. REF, homozygous reference; HET, heterozygous; ALT, homozygous alternative. **c** Distribution in each sample of 21,969 loci of scaffold3 out of 350,393 loci shown in b. The bin width is 500,000 bp.

**
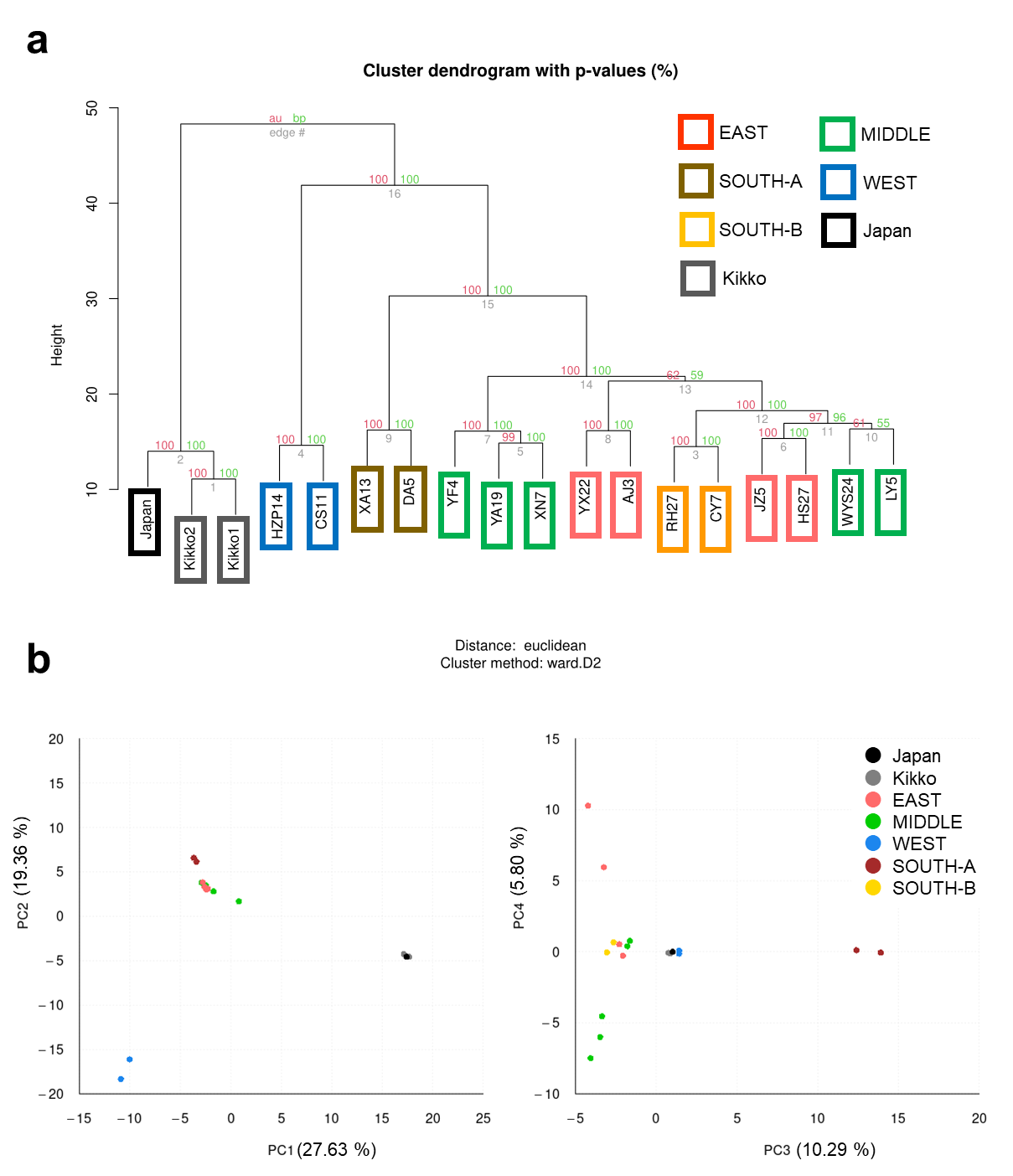
Figure S10.** Clustering and PCA could not deduce the origin of Japanese Moso bamboo. The 9,104 loci used in Fig. 2a were analyzed. Genotype was converted to homozygous reference=0, heterozygous=0.5, homozygous alternative=1. **a** The clustering was performed using pvclust of R package. The analysis condition is Euclidean distance, Ward.D2 method, nboot=1000. **b** PCA was performed using the R function prcomp.


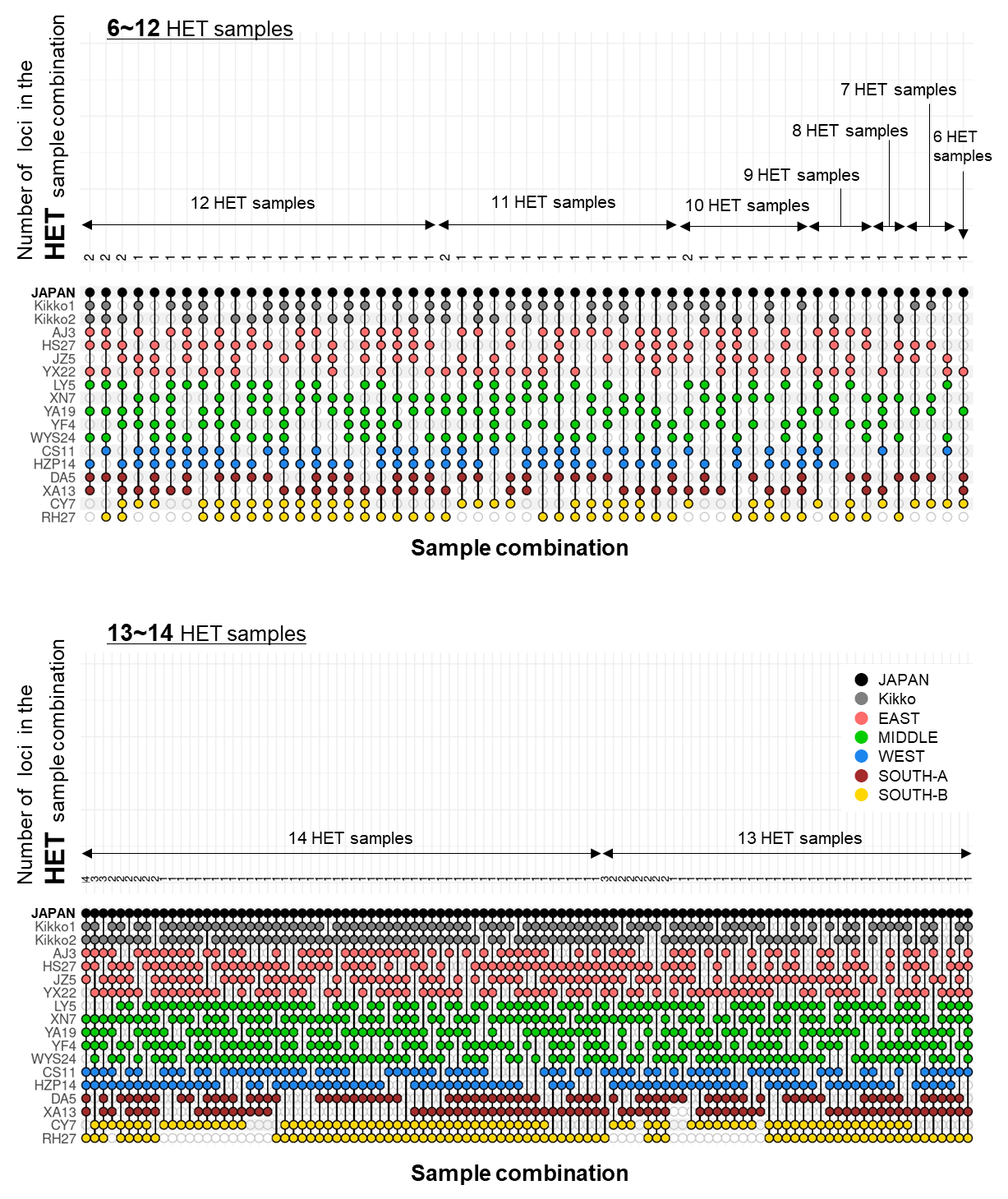


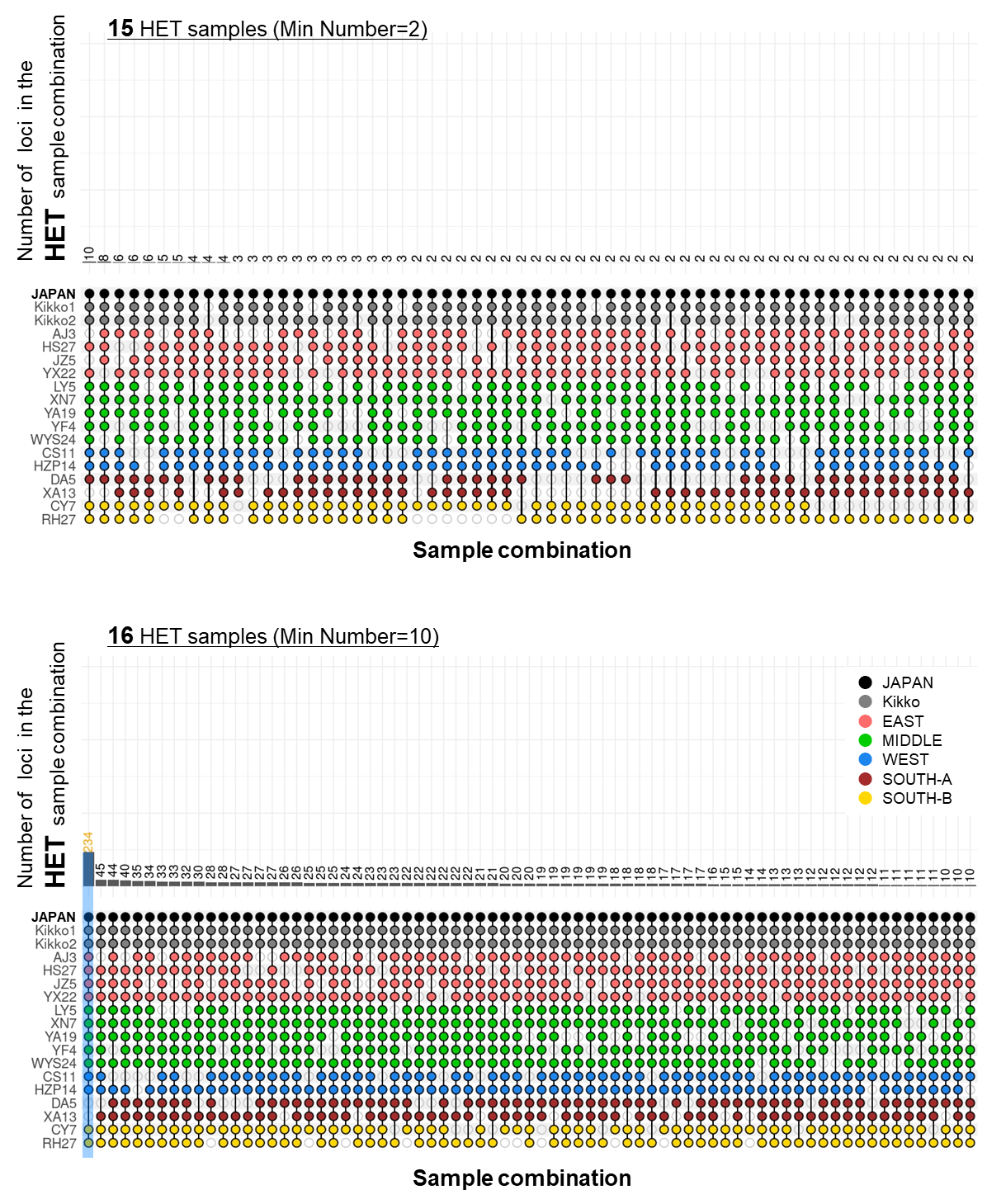


**Figure S11.** Analysis of the origin of Japanese Moso bamboo by analysis of heterozygous SNPs common among samples. The selected 4,750 SNPs shown in fig. 2a were analyzed using UpSet plots. UpSet plots are alternative to the Venn Diagram used to deal with more than 3 sets. These figures were created using an R package called ComplexUpset. Analysis was performed under the condition of “sort_intersections_by = c (‘degree’,‘cardinality’)”. For sample combinations other than 15 and 16, all sample combinations are shown. For sample combinations with 15 heterozygous samples, only sample combinations with the minimum number of loci of 2 or more than 2 are shown. For sample combinations with 16 heterozygous samples, only sample combinations with the minimum number of loci of 10 or more than 10 are shown. HET, heterozygous.


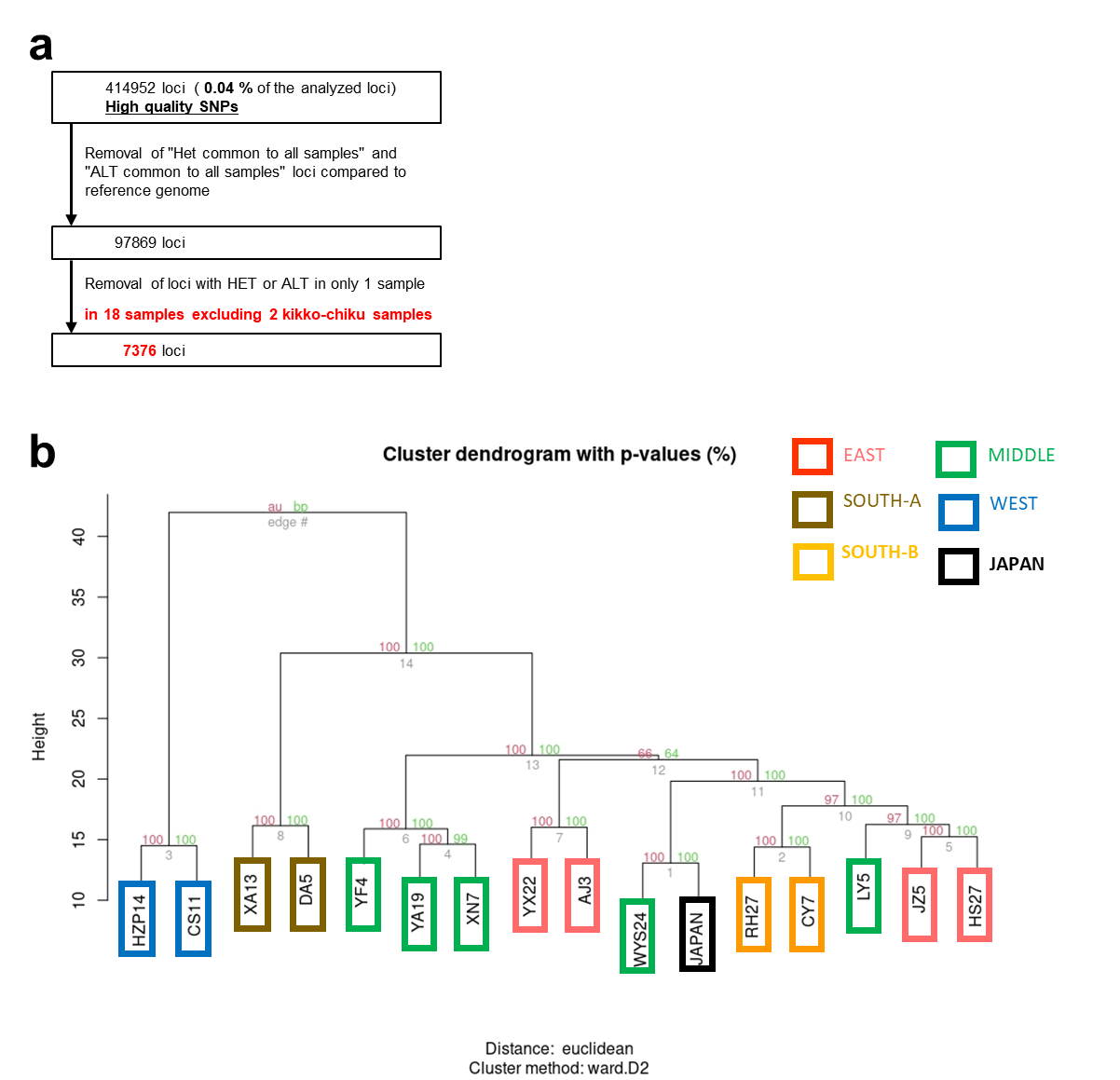


**Figure S12.** Clustering analysis without 2 kikko-chiku samples. **a** With the exclusion of the 2 samples of Japanese Kikko-chiku, loci with no polymorphism among samples or one sample having a different genotype from the others were excluded. **b** The 7,376 loci were analyzed. Genotype was converted to homozygous reference=0, heterozygous=0.5, homozygous alternative=1. The clustering was performed using pvclust of R package. The analysis condition is Euclidean distance, Ward.D2 method, nboot=1,000.


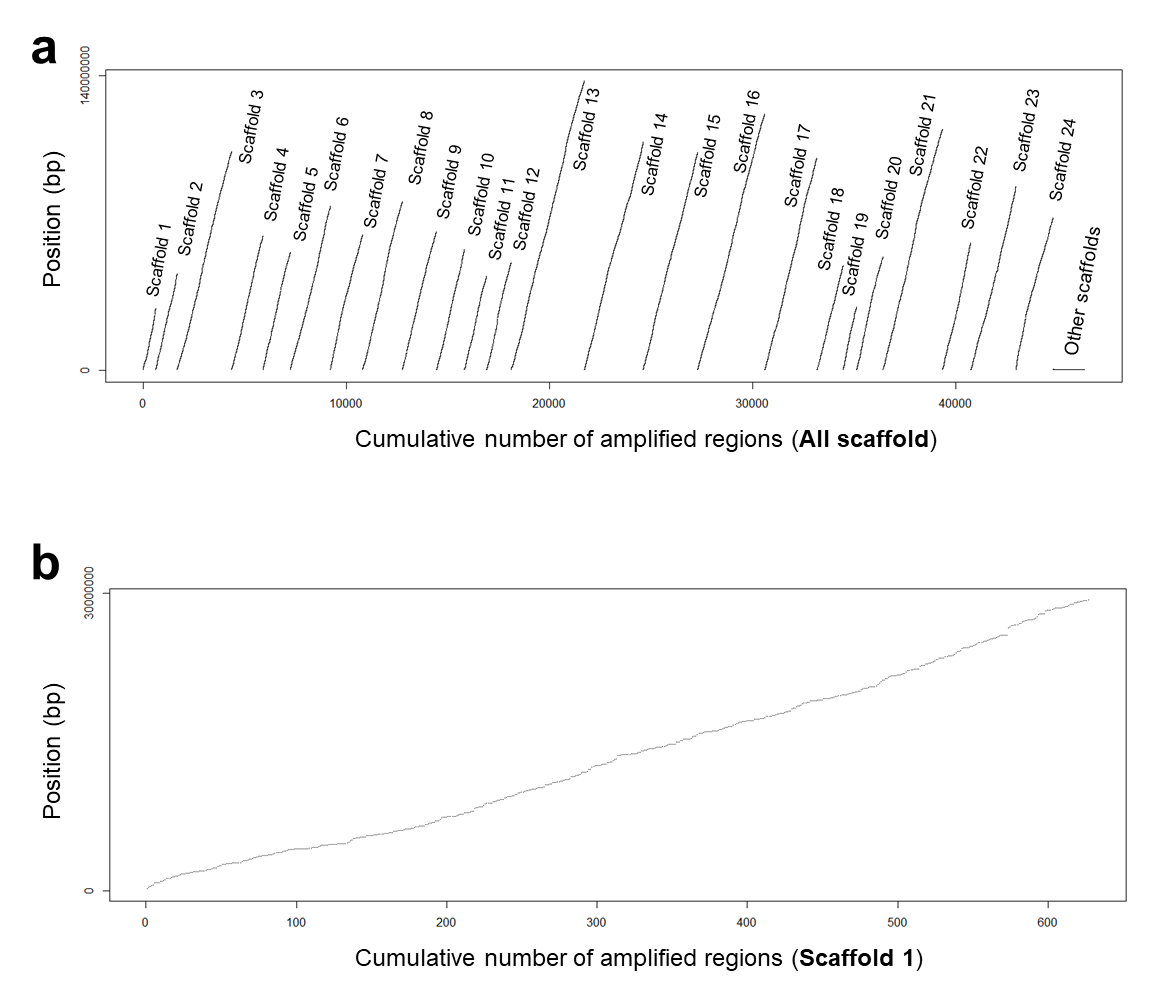


**Figure S13.** Partial sequences of the Moso bamboo genome were evenly amplified from the genome using GRAS-Di. We plotted the tip loci of each amplified region with a depth of 10 or more for all 16 samples. **a** All scaffolds. **b** Scaffold1.


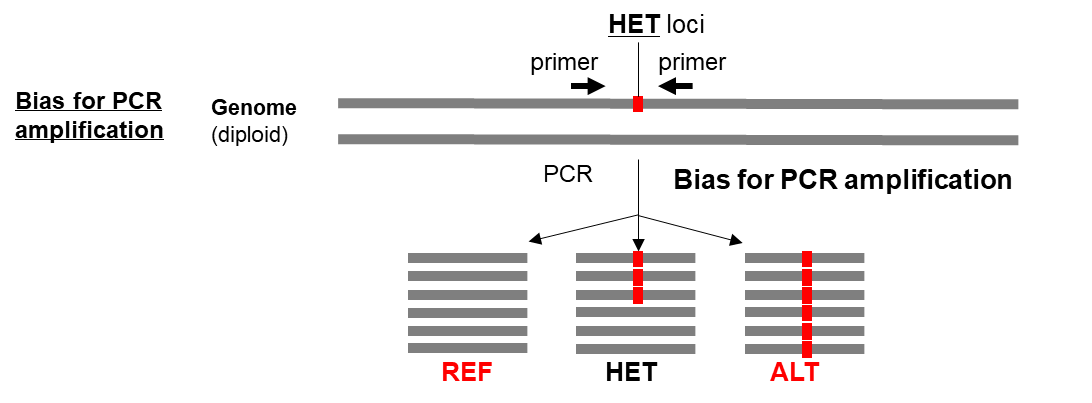


**Figure S14.** A model in which heterozygous loci are falsely detected as different genotypes in GRAS-Di analysis. Red letters indicate incorrect genotyping. REF, homozygous reference; HET, heterozygous; ALT, homozygous alternative.


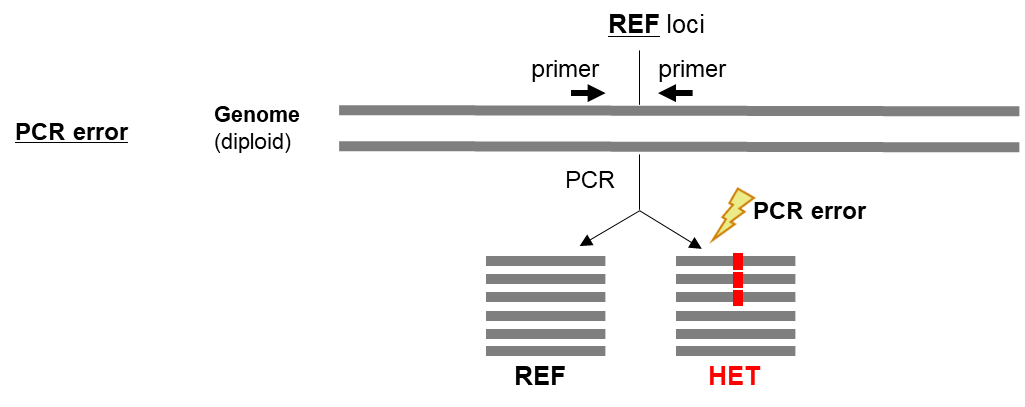


**Figure S15.** A model in which REF loci are falsely detected as HET in the GRAS-Di analysis. This model is a model in which PCR error causes incorrect genotyping. Red letters indicate incorrect genotyping. REF, homozygous reference; HET, heterozygous.


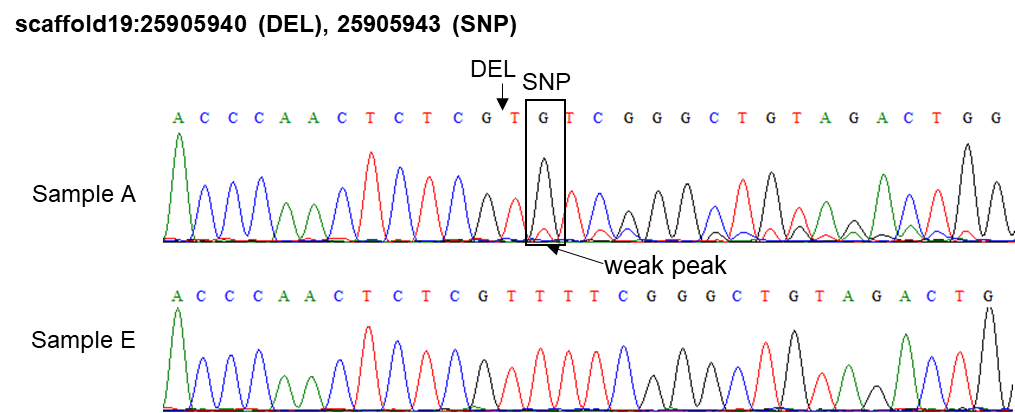


**Figure S16.** Among the homozygous alternative loci of Moso bamboo, there were loci that may be chimeras. The loci in this figure, which were determined to be heterozygous by GRAS-Di analysis, were checked by Sanger sequencing and were thought to be homozygous alternative, but could be chimeras. In the 2 homozygous alternative loci, weak reference sequence signals were also visible, suggesting chimeras.


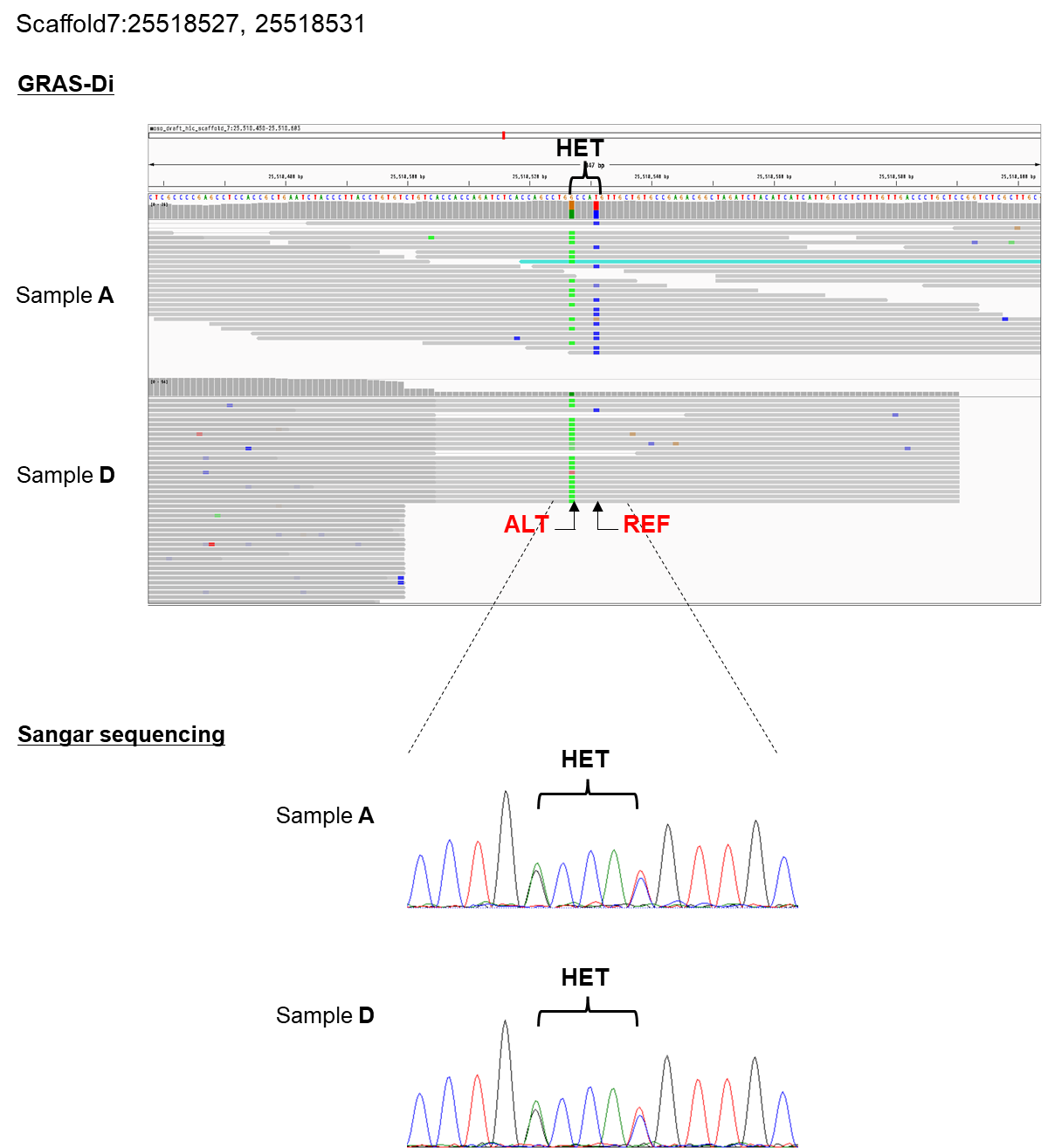


**Figure S17.** In the GRAS-Di analysis, incorrect genotyping occasionally occurred due to bias during PCR. This figure shows as an example of the model shown in Additional file1: Fig. S14. In the GRAS-Di analysis, genotypes 2551827 and 25518531 of Scaffold7 in Sample D were determined inaccurately due to bias during PCR. The upper half figure shows the results of GRAS-Di amplified sequences with IGV. The lower half of the figure shows the result of Sangar sequencing. Scaffold7:25518527 in Sample D was ALT and scaffold7:25518531 was REF in the GRAS-Di analysis, but both loci were heterozygous in the Sangar sequencing. Red letters indicate incorrect genotyping. REF, homozygous reference; HET, heterozygous; ALT; homozygous alternative.


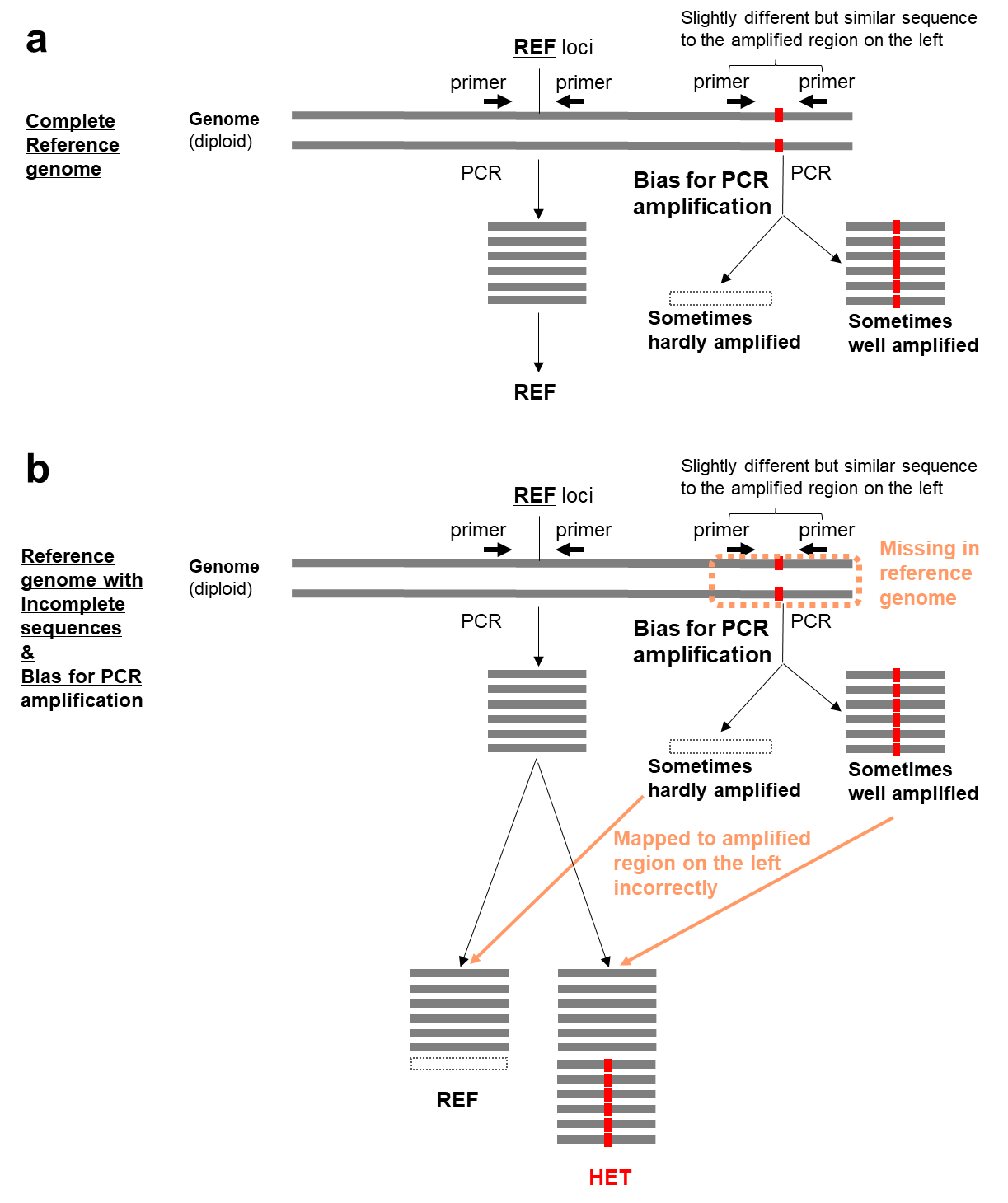


**Figure S18.** A model in which REF is falsely detected as HET in the GRAS-Di analysis. **a** Homozygous reference loci is correctly determined to be a homozygous reference if the reference genome is complete. **b** This figure shows the case where some sequences of the genome are missing from the reference genome. If the missing sequence in the reference genome is amplified or not due to bias during PCR, the homozygous reference loci may be correctly determined to be homozygous reference, but may also be determined to be HET due to incorrect mapping. Red letters indicate incorrect genotyping. REF, homozygous reference; HET, heterozygous.


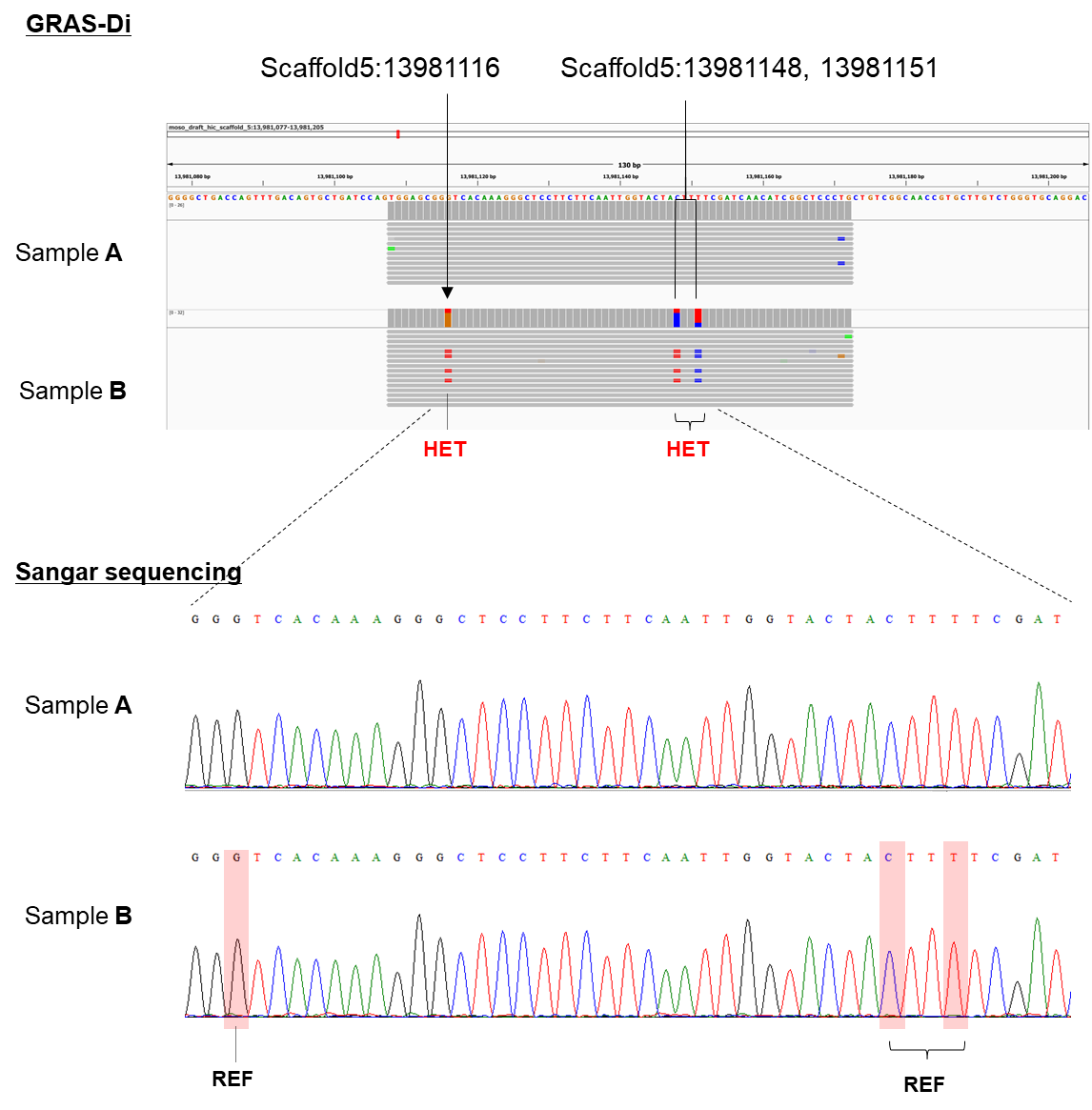


**Figure S19.** In the GRAS-Di analysis, homozygous reference loci were occasionally incorrectly determined to be heterozygous loci. In GRAS-Di analysis, genotypes of 13981116, 13981148, and 13981151 of Scaffold5 in Sample B were all heterozygous, but those loci were all homozygous reference in Sanger sequencing. In the GRAS-Di analysis, 13 samples of 13981116 from Scaffold 5 and 14 samples of 13981148 and 13981151 from Scaffold 5 were incorrectly determined as heterozygous. In the PCR error model in Additional file 1: Fig. S15, it is unlikely that the same error would occur in the same loci of a large number of samples. Probably, these errors were caused by "incomplete reference genome and bias during PCR", which corresponds to Additional file 1: Fig. S18b. REF, homozygous reference; HET, heterozygous.
